# Design and implementation of a graphene–polyimide-based H-slot terahertz antenna for wireless and biomedical applications

**DOI:** 10.1371/journal.pone.0336921

**Published:** 2025-12-01

**Authors:** K. V. Vineetha, M. Siva Kumar, B. Sada Siva Rao, Om Prakash Kumar, Sudha Rani V, B. T. P. Madhav

**Affiliations:** 1 Antennas and Liquid Crystals Research Center, Department of ECE, Koneru Lakshmaiah Education Foundation, Guntur, AP, India; 2 Dept of ECE, Swarnandhra College of Engineering and Technology (A), Narsapuram, West Godavari, AP, India; 3 Department of Electronics and Communication Engineering, Manipal Institute of Technology, Manipal Academy of Higher Education, Manipal, 576104, India; 4 School of CS&AI, SR University, Warangal, Telangana, India; Parul University Parul Institute of Technology, INDIA

## Abstract

THz antennas, which function at high speeds, frequencies, and data rates, were developed in response to the increased need for high-speed communication equipment. In this work, a MIMO antenna operating between 2.25 and 2.85 THz is built and optimised with partial ground. The designed antenna possesses H-shaped slots and circular rings over the patch to enhance the antenna performance. The proposed antenna states the isolation loss value of 50 dB across the operating frequency with a bandwidth of 0.6 THz. In the manuscript, two antennas were designed, the first one having only circular slots and the second one, in addition to the circular slots over the patch, also including H-slots. The antenna has the highest gain value of 8.9 dBi in the design. Optimising the design is performed using parametric optimisation and geometrical parameters. The suggested antenna measures 50 x 50 x 100 µm². The suggested antenna can be used for high-speed communications because of its high gain and operating frequency applicability. Antenna having a low Error Correlation Coefficient (ECC) value of 0.08, a high Diversity Gain (DG) value with minimum mutual coupling < -25dB, an optimum Total Active Reflection Coefficient (TARC) value of -55dB and a Mean Effective Gain (MEG) value of 8.5dB. These antennae also operate across biomedical imaging applications, wireless network applications, beam scanning applications, and satellite communication applications with reflection coefficient values < -25dB.This study supports UN SDG 9: Industry, Innovation and Infrastructure by advancing sustainable THz communication technologies, and contributes to SDG 3: Good Health and Well-Being through its biomedical imaging applications.

## 1. Introduction

Demands on cellular data traffic have skyrocketed as a result of the marked growth in data use, which includes streaming HD video, playing real-time online games, and watching live sports. 18.22 billion mobile devices will have internet access by 2025. 5G and 6G will offer a speed of 20 Gbps and 1 terabit per seconds respectively. For 1.2 terabits, the frequency range is 300 GHz to 10 THz (1 THz = 1012 Hz). THz waves are thought to have a bandwidth of more than 10 GHz [1–3] between millimetre waves and infrared light waves. THz technology could be able to handle the enormous demand for mobile data traffic as data rates increase from Gbps to Tbsp. Even though Tbsp. is now a hot research issue, we have achieved Gbps rate of data in the current enivornment [4,5]. A future wireless communications and security system will incorporate terabits per second (Tbsp.) wireless local area networks (Tera-WiFi), THz cellular connections, on-chip communication, remote sensing, THz wireless personal area networks (T-WPAN), THz Internet of Things (Tera-IoT), THz integrated access backhaul (Tera-IAB) wireless networks, THz chemical imaging, THz imaging for defence and security transceivers, THz cameras, and THz imaging. The THz band may find application in high-resolution radar [6,7]. First-generation (1G) technologies, which were based on analogue systems and gained popularity in 1980, marked the beginning of mobile wireless technology. Nordic Mobile Telephone (NMT), Advanced Mobile Phone System (AMPS), and Total Access Communications System are examples of 1G systems. The foundation of second-generation (2G) networks is digital technology. In addition to IS-95/CDMA, 2G technologies include the Global System for Mobile Communications (GSM). A 2G system could only support text and voice calls. The term 2.5G, or greater data rates, refers to the expansion of 2G GSM evolution (EDGE) for low-speed internet and phone services [8,9]. CDMA 2000 and Wideband-CDMA are the 3G interfaces. As a result of the introduction of 3G, the era of smartphones, with actual mobile data and internet surfing, became a reality. The range of frequencies of 1.8–2.5 GHz is used by 3G to offer data speeds of up to 2 Mbps. Network coverage issues and capacity requirements for worldwide usage of 3G have been observed [10,11]. 4G wireless networks are more efficient than earlier generations, making them the most advanced wireless broadband systems available. In 4G, the access technology used is OFDMA. Online gaming and the downloading and uploading of high-definition (HD) videos are supported by 4G. A 20 Mbps of data is supported with 4G [12,13]. Numerous services, including as device-to-device communication, cloud-based live gaming, smart cities, smart homes, and industrial automation, are not compatible with 4G networks at high data rates. As a result, 5G networks are very desirable due to their enormous data capacity, dependable connectivity, and exceptionally low latency [14–17]. The study shows that incorporating Metasurfcae-inspired slot and via structures between antenna elements effectively minimizes mutual coupling, suppresses substrate and surface-wave losses, and enhances the overall performance of MIMO arrays—without increasing the antenna’s physical size. The proposed technique is simple, symmetric, and scalable, providing an excellent balance between design complexity and performance improvement when compared to more sophisticated decoupling methods such as electromagnetic bandgap structures, defected ground planes, or metamaterial resonators. However, the validation is limited to a 2 × 2 array, and its applicability to larger configurations (such as 4 × 4 or 8 × 8 arrays) or to more closely spaced elements remains an open area for future investigation [18]. The proposed antenna design effectively tackles key limitations of on-chip terahertz antennas by integrating substrate-integrated waveguide (SIW) confinement with Metasurfcae-based enhancements and a backside electromagnetic coupling feed. It delivers a comparatively broad bandwidth and reasonable radiation efficiency for on-chip THz operation, all while maintaining a compact physical footprint. The underside feeding approach further facilitates seamless circuit integration by eliminating the need for intricate feed routing on the radiating surface. Nevertheless, certain challenges persist — silicon substrate losses are only partially mitigated, precise fabrication of the multilayer structure and slot geometries remains critical, and extending the design to larger antenna arrays for beamforming could introduce additional coupling complexities [19]. The study shows that integrating Metasurfcae-inspired slotting with electromagnetic bandgap (EBG) decoupling results in a compact, wideband MIMO antenna array offering excellent isolation—well suited for wearable and biomedical telemetry applications. A notable strength of the work is the inclusion of bending and deformation analyses, which confirm the antenna’s resilience under practical mechanical stresses. The design achieves a balanced compromise among isolation, gain, efficiency, and compactness. However, its implementation is currently limited to a 2 × 2 configuration; extending the approach to higher-order MIMO systems or tighter element spacing requires additional research. Furthermore, the influence of human-body proximity—such as detuning and absorption effects—warrants more detailed investigation [20]. The developed configuration combining a swiveled dielectric resonator antenna (DRA), substrate integrated waveguide (SIW), and embedded photonic band gap (PBG) structure effectively achieves tri-band terahertz operation with notable gain and radiation efficiency within a compact micrometre-scale design. The inclusion of a metal-free PBG guiding layer proves advantageous in the terahertz domain by minimizing propagation losses and enhancing wave confinement. Such an antenna structure holds strong potential for use in next-generation 6G systems, high-speed THz communication, terahertz imaging, sensing, and IoT applications. For future research, the authors propose investigating alternative low-loss dielectric materials—such as high-resistivity silicon, quartz, and polymer substrates—to further improve overall performance and efficiency [21]. The study introduces a four-port MIMO patch antenna featuring a wheel-shaped structure, optimized for ultrawideband (UWB) functionality within the terahertz (THz) frequency range. It is specifically developed for 6G communication systems, which demand high data throughput and efficient utilization of THz spectrum. The proposed design emphasizes achieving broad bandwidth, strong port isolation, and a compact configuration suitable for seamless integration into advanced THz devices [22]. The study presents a multiband MIMO antenna optimized for UAV communication across millimetre-wave and sub-terahertz bands, offering enhanced gain, high efficiency, stable circular polarization, excellent port isolation, and beam-steering capability. Its versatile operation supports drone-to-ground, drone-to-drone, and drone-to-satellite connectivity, positioning it as a promising solution for 6G and beyond communication networks. By integrating leaky-wave principles, meta surface structures, and precisely engineered slot and feed designs, the authors achieve a compact, planar, and easily mountable antenna suitable for UAV applications. Future work may focus on experimental verification at higher frequencies, better integration with commercial UAV platforms, and the development of adaptive or reconfigurable meta surface technologies to further enhance performance [23]. The designed graphene-assisted slot antenna presents an effective approach for achieving compact and frequency-tunable multiband THz operation, making it suitable for adaptive THz communication, sensing, and reconfigurable wireless networks. Through electrostatic biasing, the graphene layer enables dynamic frequency tuning, allowing a single antenna structure to operate across several bands without additional hardware components. However, challenges such as precise fabrication, bias circuit integration, and maintaining consistent performance under practical conditions still need to be addressed. Future studies may focus on prototype fabrication and measurement, the use of alternative graphene arrangements like patterned or multilayer structures, and the incorporation of this reconfigurable slot design into MIMO systems or beam-steerable architectures [24].

The proposed antenna design consists of H-slots improve antenna gain and isolation by suppressing surface currents and surface waves, enhancing radiation efficiency, and minimizing electromagnetic coupling between elements. The inclusion of H-shaped slots in the antenna design enhances its performance through multiple mechanisms. These slots interrupt the surface current flow between neighbouring elements, thereby minimizing mutual coupling and improving isolation. They also create localized high-impedance zones that restrict surface-wave propagation, effectively reducing interference. By suppressing surface-wave and substrate losses, more electromagnetic energy is radiated into free space, resulting in higher radiation efficiency and improved gain. Furthermore, the modified structure supports a more balanced current distribution, enhancing the overall radiation characteristics. The H-slot also behaves as a secondary resonator, generating out-of-phase fields that help neutralize coupling energy and further strengthen antenna isolation.

## 2. Proposed antenna modelling and methodology

### 2.1 Material characteristics of the substrate

In antenna design, polyimide (PI) substrates are frequently used over substitutes such as PTFE (Teflon), PEN (Polyethylene Naphtholate), LCP (Liquid Crystal Polymer), PET (Polyethylene Terephthalate), and quartz because of a number of significant benefits. Because of these benefits, polyimide substrates are especially well-suited for high-performance and flexible antenna applications in Table 1. This explains why polyimide substrates are better.

**Table 1:** Comparison of several substrates for THz dielectrics.

Variable	PEN	PET	LCP	PTEF (Teflon)	Quartz	Polyimide
Dissipation factor (tan^5^)	4.8e^-3^	1.1e^-3^	2.5e^-3^	2e^-3^	2e^-3^	2.7e^-3^
Thermal expansion co-efficient (1e^-6^/K)	20	60	0-30	60	140	7.50
Density (Kg/m^3^)	1360	1380	1760	1380	2200	1400
Dielectric constant(€_r_)	3.2	6.4	2.90	1.96-2.1	3.05	2.3-13.4
Thermal conductivity (W/m.K)	0.15	0.28	0.25	0.28	0.2	0.04-1.7
Youngu’s Modulus (GPa)	5.5	1	2-10	1	0.5	0.5

**Mechanical Properties and Flexibility**: Even under severe mechanical stress, such as bending and folding, polyimide, which is well known for its remarkable flexibility and mechanical strength, is resistant to breaking and degrading. This makes it perfect for conformal antennas, where the material needs to be bent or moulded and Internet of Things applications. Whereas PEN/PET Particularly at high temperatures, they are less structurally strong and flexible than polyimide. Similarly, LCP Generally, more brittle than polyimide, LCP also provides significant flexibility.

**Stability of Heat:** Because of its exceptional thermal stability and ability to perform in a broad temperature range (−200°C to 400°C), polyimide is a good choice for high-temperature applications like tough industrial settings or space applications. On other hand side LCP although it can only withstand temperatures lower than those that polyimide can withstand, LCP likewise has high thermal stability. Similarly, PEN/PET these substrates are more susceptible to heat deterioration and have less thermal stability. Teflon, also known as PTFE, is more challenging to produce and lacks mechanical flexibility while having strong heat resistance. Although extremely thermally stable, quartz is stiff and inappropriate for flexible antenna designs.

**Properties of Dielectrics**: Polyimide is appropriate for RF/microwave antennas due to its moderate dielectric constant (usually between 3.0 and 3.4) and minimal dielectric losses at high frequencies. Antenna performance is maintained with little signal loss because to this balancing. On other hand side at higher frequencies, PEN/PET antennas may perform worse due to their increased dielectric losses. Similarly, Although LCP has a low dielectric constant (around 2.9), making it ideal for radiofrequency applications, processing and bonding with other materials can be more challenging. In addition to this Teflon, or PTFE, is a material that is good for high-frequency applications due to its extremely low dielectric constant (~2.1). However, its usage in some designs is limited by its difficulty in thin-film manufacturing. Likewise, although quartz has a relatively low dielectric constant, many antenna applications cannot use it due to its rigidity and expense.

**Chemical Resistance**: Polyimide due to its strong chemical resistance, polyimide can be used in difficult environments where exposure to oils, solvents, or other chemicals may be problematic. On other hand side Compared to polyimide, PEN/PET have lower chemical resistance. Similarly, Although LCP has strong chemical resistance, its long-term dependability may be impacted by its propensity to absorb moisture. Although Teflon, or PTFE, has outstanding chemical resistance, its mechanical characteristics make it less suitable for flexible designs.

**Processing and Thin Film Capabilities**: Polyimide is necessary for lightweight, small, as well as flexible antenna designs since it can be produced as extremely thin sheets, even smaller than 25 microns. Additionally, it works with common photolithographic techniques, which makes integrating it into sophisticated antenna manufacture simple. On other hand side Although LCP it is often more costly and difficult to manufacture, LCP may also be produced in thin films. Similarly, although thin films of PEN and PET substrates are available, they lack the mechanical strength and thinness of polyimide. In addition to this PTFE and quartz are stiff materials that aren’t usually made into ultra-thin films for flexible uses.

**Price and Availability:** Although it is not the most affordable material, polyimide provides a decent trade-off between price and performance, especially in demanding applications. On other hand side PEN/PET are often less expensive, but their flexibility, thermal stability, and dielectric properties are noticeably inferior. Similarly, compared to polyimide, LCP is usually more costly and scarcer. In addition to this Quartz and PTFE are frequently more expensive and impractical for large-scale manufacturing, especially in flexible or small-sized systems.

Flexibility, thermal stability, strong dielectric qualities, chemical resistance, and simplicity of processing, polyimide substrates are more beneficial in antenna design than PEN, LCP, PET, Quartz, and PTFE (Teflon). Because of these qualities, polyimide is perfect for high-performance antennas, especially in applications requiring flexibility, compactness, and severe environments where other materials would not work as well or at all.

While both Polyethylene Naphtholate (PEN) and Polyimide (PI) offer desirable dielectric and mechanical properties for flexible antenna substrates, Polyimide is more suitable for operation at millimetre-wave and terahertz frequencies. Its lower dielectric loss tangent, enhanced thermal and chemical stability, and smoother surface profile help reduce signal attenuation and support high-quality metallization. In addition, Polyimide demonstrates stable permittivity and minimal frequency dispersion across wide spectral ranges, which ensures consistent impedance and phase characteristics. Consequently, Polyimide serves as a more efficient substrate choice for advanced high-frequency and terahertz antenna systems compared to PEN.

### 2.2 Conductive materials for radiating element

High electrical and thermal conductivity, flexibility, low weight, High frequency range operation, stability in chemical and transparency make graphene superior than copper and carbon nanotubes in many antenna and electronic design applications. Copper is a reliable, versatile, and lightweight material, but it is not appropriate for high-frequency, modern applications. Graphene is the more attractive hybrid material for future generation including 5G antennas, wearable technology, and space electronics since CNTs have certain characteristics with graphene but have problems with cost, homogeneity, and scalability which states in Table 2.

**Table 2:** Comparative evaluation of several THz antenna conductive material qualities.

Variable	Copper	Graphene	Carbon Nano tubes
Mobility of electronics	32 cm^2^ V^1^ S^1^	2x10^5^ cm^2^ V^1^ S^1^	8x10^4^ cm^2^ V^1^ S^1^
Density of current	10^6^ Acm^-1^	10^9^ Acm^-1^	10^9^ Acm^-1^
Durability	587 MPa	1.5 TPa	5-500 GPa
Diffusivity	400 W m^-1^ K^-1^	5000 W m^-1^ K^-1^	3000 W m^-1^ K^-1^

Graphene has garnered interest as an antenna and electrical application material due to its many benefits over carbon nanotubes (CNTs) and copper. The following explains why graphene is frequently thought to be more beneficial in particular applications.

**Elevated electrical conductivity:** Graphene because of its unique atomic structure, a single layer of carbon atoms organised in a hexagonal lattice Graphene exhibits extraordinarily high electrical conductivity. Graphene is perfect for high-speed electronics and high frequency because electrons can pass through it with very little resistance, especially at ambient temperature. For high-frequency antenna designs, it facilitates ballistic transit of electrons, which allows them to go farther without scattering. On other hand although copper has a high conductivity, it has a greater electron scattering rate, particularly at tiny scales. Copper has a skin effect in high-frequency applications, which causes resistance to rise and power losses since current only flows on the surface. In addition to this because it is difficult to align the tubes correctly, carbon nanotubes (CNTs) have a lower bulk conductivity than graphene, while having high conductivity as well, particularly when single-walled. Defects and contaminants can also reduce CNTs’ conductivity in large-scale applications.

**Mechanical Strength and Flexibility**: One atom thick, graphene is very thin, flexible, and mechanically robust. Its tensile strength is 100 times that of steel. Because of this, it is perfect for wearable antennas, flexible electronics, and other applications where materials must bend or adapt to different surfaces without losing functionality. Because copper is a hard metal, it cannot be utilised for wearable or flexible electronics unless small layers are applied, which might render the metal brittle and prone to breaking. Although CNTs are robust and flexible, aggregation problems and challenges with consistent manufacture and processing make them difficult to use as a large-scale material.

**Large Surface Area:** Graphene’s surface area-to-volume ratio is unusually high due to its two-dimensional nature. This is beneficial for antennas and sensors because more surface area improves interactions with electromagnetic waves, improving the efficiency of signal transmission and reception. On other hand Copper has a lower surface area-to-volume ratio since it is a 3D substance. Because of its lesser surface interaction, copper may not perform as well as graphene in miniature antennas or sensors. Despite having a large surface area, CNTs don’t always work as well as graphene in real-world applications because to issues with alignment and organisation.

**Weight:** Graphene of its atomic structure, graphene is very light. An important benefit in applications like satellites, portable electronics, and aircraft is the significant weight reduction that graphene may provide when used in antennas and other electrical devices as compared to metals like copper. Copper is less appropriate for situations where weight reduction is crucial since it is heavier and denser. Compared to graphene, CNTs are less feasible for widespread antenna usage because to issues with integration and large-scale uniformity, despite their similar lightweight nature.

**Bandwidth and Frequency Reactivity**: Graphene’s ability to function well at higher frequencies, such as millimetre-wave and terahertz ranges, is essential for 5G and other next-generation communication technologies. It is perfect for wideband and reconfigurable antennas because of its adjustable electronic characteristics, which enable it to be tailored for various frequency bands. Although Copper works well at lower frequencies, but because of parasitic effects, skin effect, and greater losses, its effectiveness decreases at higher frequencies. In addition to this although chirality variations and the difficulty of building large-scale, defect-free CNT networks make it more difficult to control their electronic properties, CNTs also have the potential for high-frequency applications.

**Conductivity of Heat**: Graphene in high-power applications where heat control is crucial, graphene’s remarkable thermal conductivity—better than copper—is advantageous. Graphene-based antennas with effective heat dissipation are more dependable and long-lasting because they are less likely to overheat. Although graphene outperforms copper in situations where sustaining performance at high power is crucial, copper still has strong thermal conductivity. In addition, CNTs have a respectable thermal conductivity, flaws and alignment problems might lessen their usefulness in large-scale applications, much like with electrical characteristics.

**Optical Properties and Transparency**: Graphene’s optical transparency makes it perfect for uses such as optical devices and transparent antennas. Metals like copper or carbon nanotubes cannot match its unique mix of electrical conductivity and transparency. Because copper is opaque, it cannot be utilised in antennas or transparent electronics. Although CNT films are typically more resistant than graphene, they may still be produced transparent, which limits their applicability in conducting material which is transparent.

**Resistance to Corrosion and Chemical Stability:** Graphene is extremely corrosion-resistant and chemically stable. Graphene antennas have a longer lifespan than copper-based ones in hostile environmental contexts, such as industrial or marine environments. Over time, copper’s performance may deteriorate due to its susceptibility to oxidation and corrosion, particularly in moist or salty settings. Although CNTs are similarly corrosion-resistant, their lack of large-scale homogeneity still poses problems, and they can deteriorate in specific chemical conditions.

**Fig 1:**
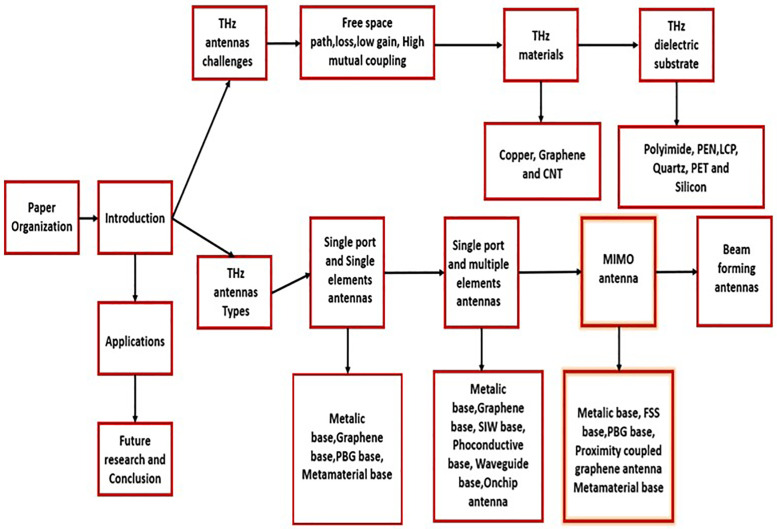
Organizational structure of the paper highlighting THz antenna types, materials, challenges, and design evolution from single-element to MIMO and beam-forming configurations.

**Scalability and Cost**: Research is moving quickly to produce large-scale, high-quality graphene sheets, even though graphene synthesis is still in its infancy. Graphene is becoming increasingly viable for commercial uses as its cost continues to decline. On other hand Copper is a reasonably priced and well-established material. However, graphene’s better qualities can make up for its greater price in high-performance and small-sized applications. In addition to this, Reliability in electronics and antennas depends on the quality and consistency of CNTs, which are currently costly and challenging to create at scale (Fig 1).

While CNTs excel in certain niche applications (e.g., nanoscale interconnects and sensors), graphene’s planar structure, process compatibility, and uniform electronic behaviour make it more suitable for next-generation high-frequency, terahertz, and flexible electronic systems.

The rapid evolution toward sixth-generation (6G) communication demands ultra-high data rates, massive connectivity, and low latency, which can be achieved by integrating Multiple Input Multiple Output (MIMO) technology with terahertz (THz) frequency bands. This study investigates the design and performance of THz MIMO antenna systems aimed at overcoming the challenges of high path loss, fabrication tolerances, and limited propagation range inherent to THz communication. The proposed methodology involves the optimization of antenna geometry and substrate parameters to enhance isolation, bandwidth, and gain, supported by full-wave electromagnetic simulations using CST and HFSS. Performance metrics such as envelope correlation coefficient (ECC), diversity gain (DG), and channel capacity loss (CCL) are analyzed to evaluate the system’s spatial diversity and reliability. The results demonstrate that the optimized MIMO configuration significantly improves link stability and data throughput compared to conventional single-element THz antennas. The study confirms that MIMO–THz integration is a key enabling technology for future high-capacity wireless communication systems, including 6G and beyond.

### 2.3 Proposed antenna description

**Fig 2:**
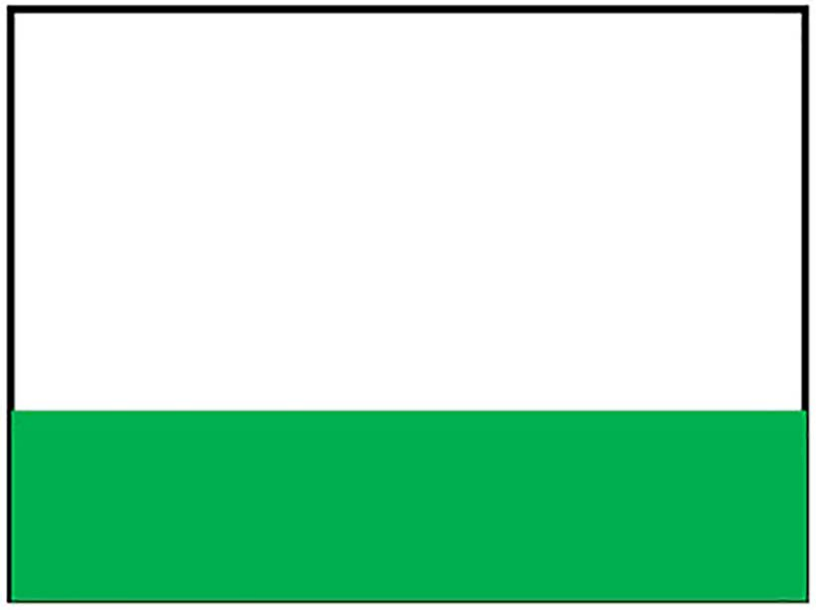
Partial ground of proposed antenna.

**Fig 3:**
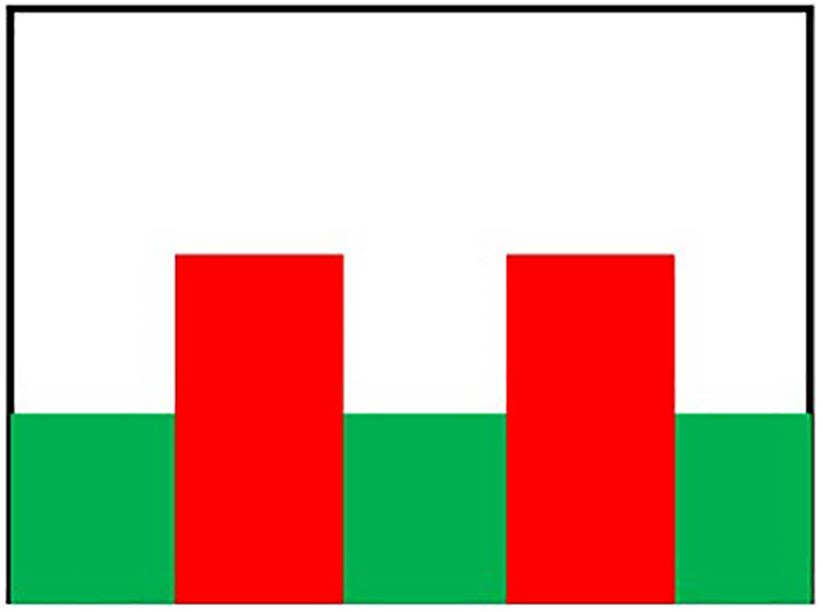
Partial ground with microstrip feed.

**Fig 4:**
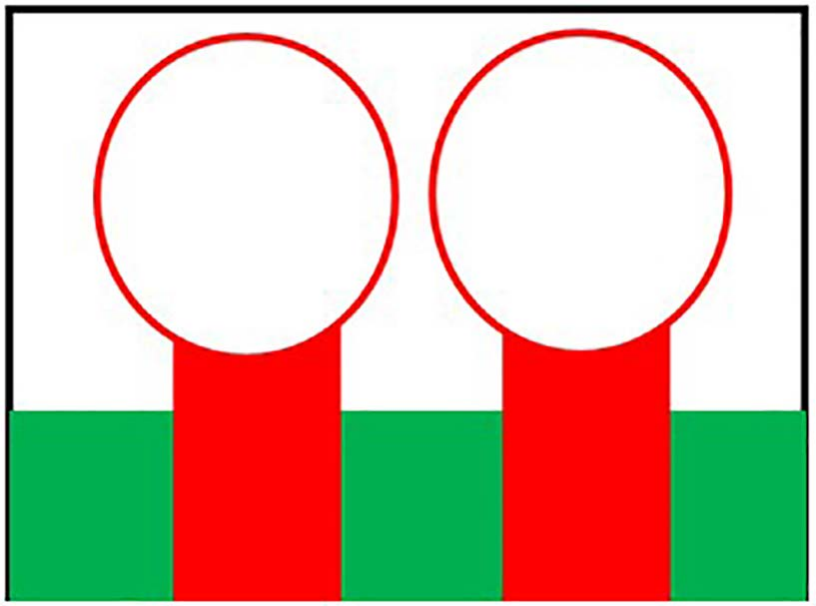
Microstrip feed with circular slot.

**Fig 5:**
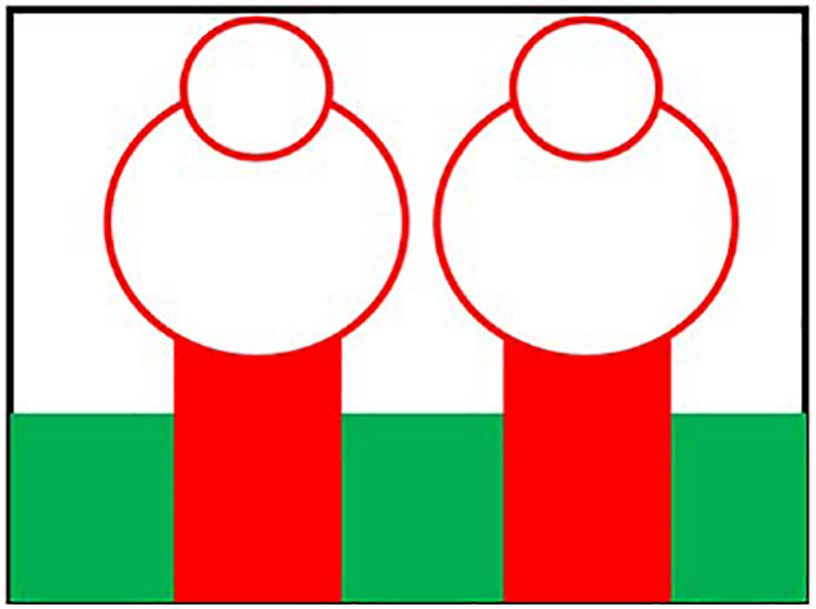
Double circular slot over the strip.

**Fig 6:**
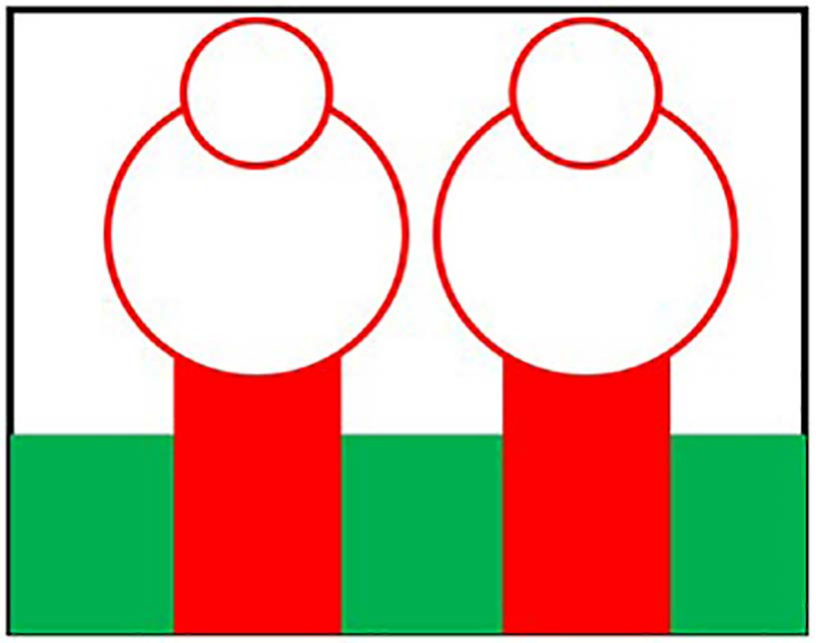
Triple Circular slot over microstrip feed.

**Fig 7:**
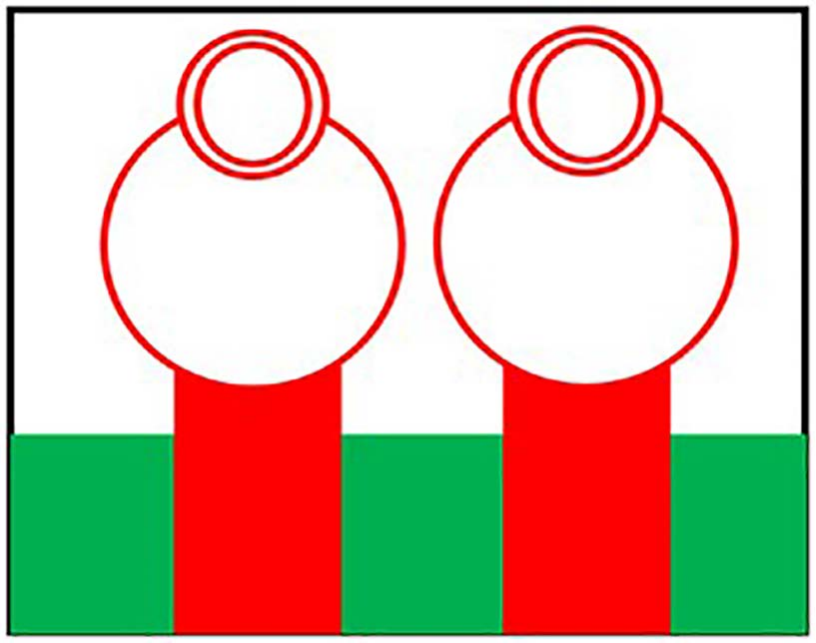
Double circular slot over Circular patch.

**Fig 8:**
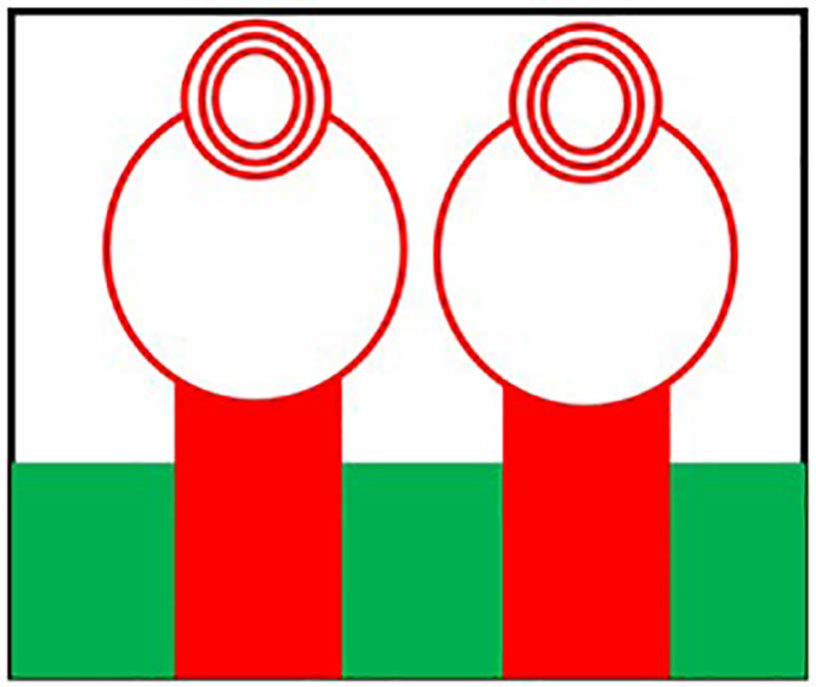
Multi Circular slot over microstrip feed.

**Fig 9:**
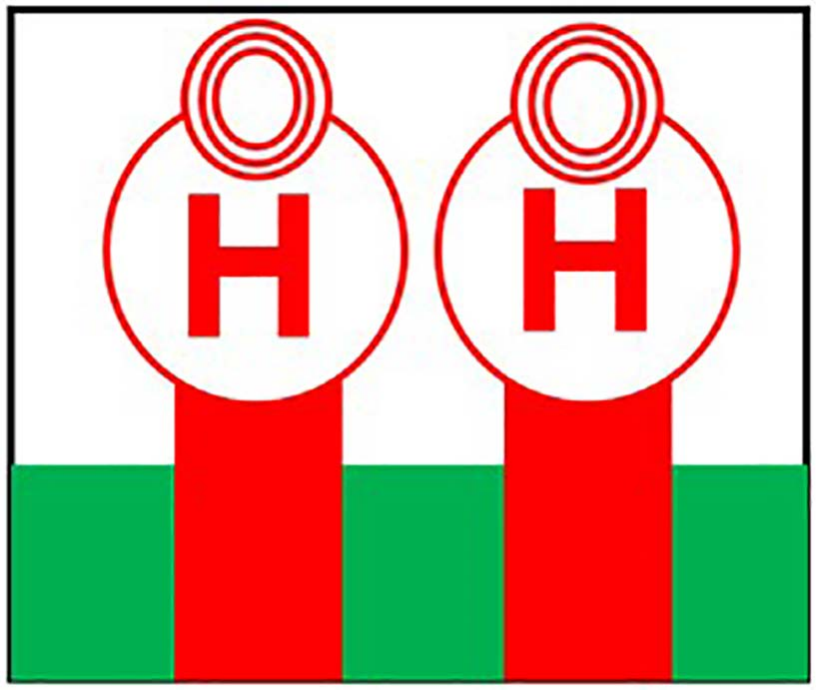
H slot inserted in Circular patch.

**Fig 10:**
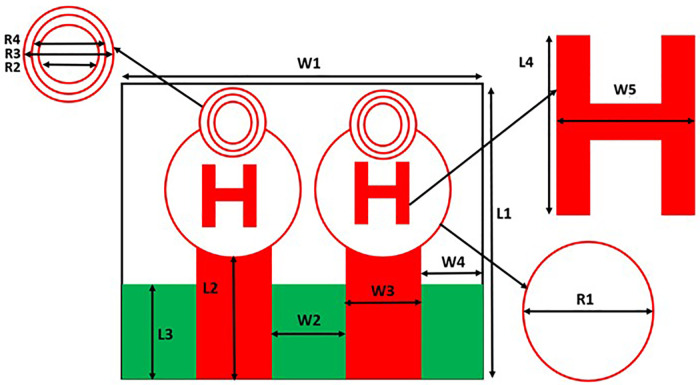
Schematic view of the proposed antenna.

**Fig 11:**
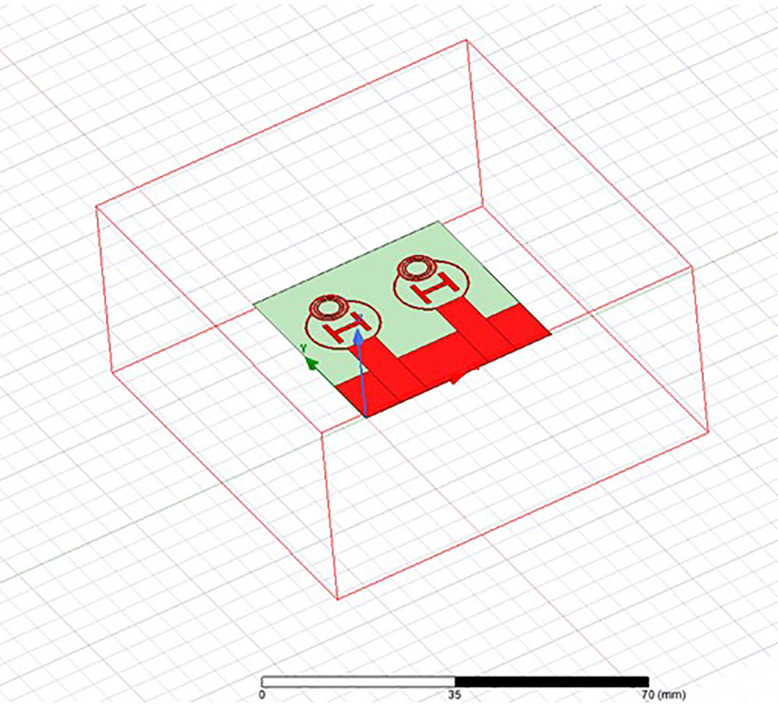
HFSS simulated design of the proposed antenna.

Initially the THz MIMO antenna is designed with simple partial ground and microstrip feed as shown in Figs 2 and 3. Later a circular slot is placed on the microstrip feed in Fig 4, then after that double circular slots are place over the strip which states in Fig 5. In addition to this triple circular slots along with H-slots are place over the patch of the antenna for performance enhancement of the proposed design which states in Figs 6–11 states the proposed antenna where three different colours are placed for better visualization of the design. The antenna’s patch is represented by the colour red, its substrate by the colour black, and its partial ground by the colour green. The suggested antenna’s substrate measures 50 x 50 x 100 µm². The antenna’s circular slot measures 8 by 10 µm². Furthermore, the antenna has a 50x12x100 µm^3^ partial ground dimension. Additionally, the substrate’s thickness is optimised to 100 µm. The proposed antenna is designed with impedance value of 50ohms. For the proposed antenna we use polyimide as a substrate having a thickness value of 0.1 mm with a dielectric constant value of 3.6. Polyimide substrates are widely used in the design and development of antennas, particularly in flexible and high-frequency applications due to its various advantages like high thermal stability, Flexibility, Low Dielectric Constant and Loss Tangent, Chemical Resistance, Lightweight, Compatibility with Additive Manufacturing, Electrical Insulation, Mechanical Strength and Environmental Stability. The antenna having polyimide as substrate material and graphene as conducting material which having huge benefits in order to enhance the performance of the antenna. The dimension of the antenna is place in Table 3.

**Table 3:** Dimensions of the proposed antenna.

Elements	Dimensions(um)	Elements	Dimensions(um)	Elements	Dimensions(um)
W1	50	L2	16	W4	8
L1	50	W3	10	L4	10
W2	14	L3	12	W5	8
R1	19	R2	10	R3	5
R4	2				

W1: This is the total width of the antenna’s radiating patch. The table indicates its dimension is 50 um.L1: This is the total length of the antenna’s radiating patch, which is also 50 um.W2: This is the width of the central connecting part of the “H” shape. The table specifies a dimension of 14 um.L2: This is the length of one of the vertical arms of the “H” shape. Its dimension is 16 um.W3: This refers to the width of the top and bottom horizontal segments of the “H” shape, with a dimension of 10 um.L3: This represents the length of the feed line that connects to the antenna, with a dimension of 12 um.W4: This is the width of the gap between the two vertical arms of the “H” structure. The table shows a dimension of 8 um.L4: This is the length of the horizontal arm of the “H” structure, which has a dimension of 10 um.W5: This refers to the width of the side-arms of the “H” structure, with a dimension of 8 um.R1, R2, R3, R4: These elements define the radii of the circular rings shown on the diagram. They are concentric circles at the top of the antenna design. Their dimensions are given as 19 um, 10 um, 5 um, and 2 um, respectively.

Equations (1)–(4) show that the length and breadth of an antenna are Vise verse proportional to the operating frequency, which is the main basis for calculating its characteristics.


$$W=\frac{V_{0}}{2F_{r}}\sqrt{\frac{\text{2}}{\varepsilon_{r}+1}}$$ (1)

W = Width of the patch (in meters), $V_{\text{0}}$= Speed of light in free space (≈ $\text{3}\times {\text{1}\text{0}}^{\text{8}}$m/s), $F_{r}$= Resonant frequency of the antenna (in Hz), $\varepsilon_{r}$= Relative permittivity (dielectric constant) of the substrate material.


$$L=\frac{C}{2F\sqrt{\varepsilon_{reff}}}-2\Delta l$$ (2)

L: The physical length of the antenna’s radiating patch.

*C*: The speed of light in a vacuum, which is approximately 3x10^8^ m/s.

*F*: The resonant frequency at which the antenna is designed to operate.

*ε*_*reff*_: The effective dielectric constant of the antenna’s substrate. The effective dielectric constant is a calculated value that accounts for the fact that the electromagnetic fields are not entirely confined within the dielectric substrate but also extend into the air.

Δ*l*: The length extension or fringing length at each end of the patch. This value accounts for the fringing fields and their effect on the antenna’s electrical length.


$$\Delta l=0.412h\frac{\left(\varepsilon_{reff}+0.03)(w+0.26h\right)}{\left(\varepsilon_{reff}-0.258)(w+0.8h\right)}$$ (3)

Δ*l*: The length extension at each end of the patch due to the fringing fields.

ℎ: The height or thickness of the dielectric substrate.

*ε*_*reff*_: The effective dielectric constant of the substrate. This value is used to account for the portion of the electromagnetic fields that travel in the air, which has a dielectric constant of 1, and the portion that travels within the substrate material itself. The value of *ε*_*reff*_ is always slightly less than the relative dielectric constant of the substrate material, *ε*_*r*_.

*W*: The width of the antenna’s radiating patch.


$$\varepsilon_{reff}=\frac{\varepsilon_{r}+1}{\text{2}}+\frac{\varepsilon_{r}-1}{\text{2}}{\left[1+\left[\frac{12h}{w}\right]\right]}^{-1/2}$$ (4)

*ε*_*reff*_: The effective dielectric constant. This value is always less than the relative dielectric constant of the substrate material.

*ε*_*r*_: The relative dielectric constant (or permittivity) of the substrate material. This is a property of the material itself and is a given value.

ℎ: The height or thickness of the dielectric substrate.

*W*: The width of the antenna’s radiating patch.

**Fig 12:**
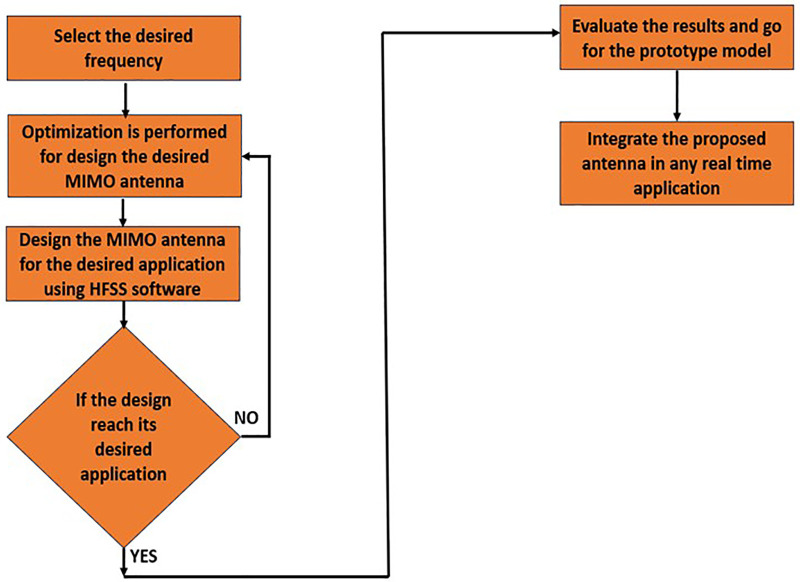
Design flow of the proposed MIMO antenna, illustrating frequency selection, optimization, simulation using HFSS, validation, and integration into real-time applications.

Fig 12 states the proposed antenna methodology which states how the dimensions of the antenna is performed and later how the optimized antenna is developed and later how it was integrated with the real time environment. Initially, we select the desired frequency where our antenna should operate. Secondly, we optimize the dimensions of the antenna by using theoretical equations. Ones the optimization was done later the antenna was designed using commercially available software, i.e., (HFSS/CST) and simulation is performed using the same software. In this step ones we get our output at desired frequency we will continue with prototype model and finally the output is verified using vector network analyser. In, case if we don’t get the desired frequency again the loop continues from parametric optimization until to get desired output.

### 2.4 Parametric analysis

Because parametric analysis enables engineers to methodically examine and optimise antenna performance by modifying critical design parameters, it plays a critical role in antenna design. This method aids in meeting the requirements for many applications, including improved bandwidth, signal strength, coverage, and overall efficiency.

**Fig 13:**
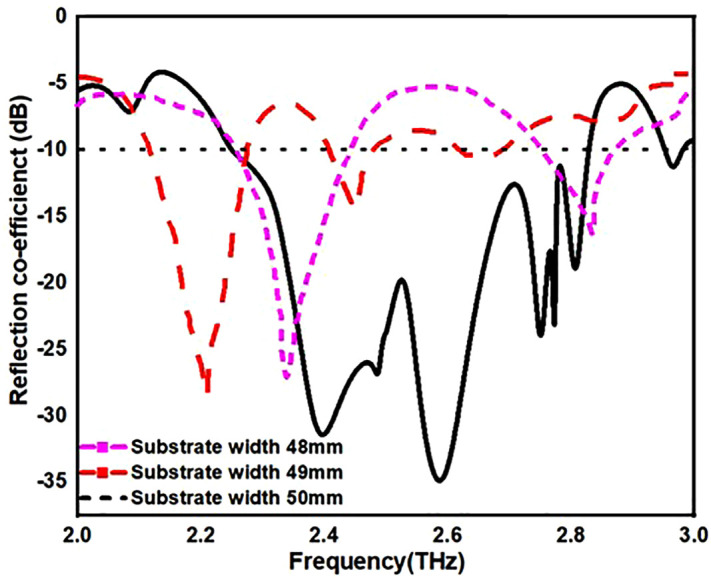
S11 value by changing substrate width.

**Fig 14:**
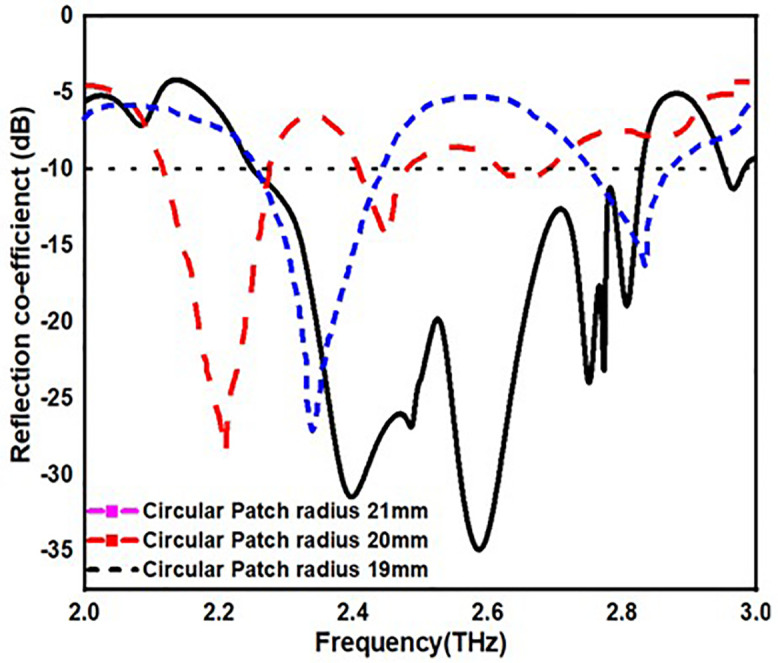
S11 value by changing patch radius.

**Fig 15:**
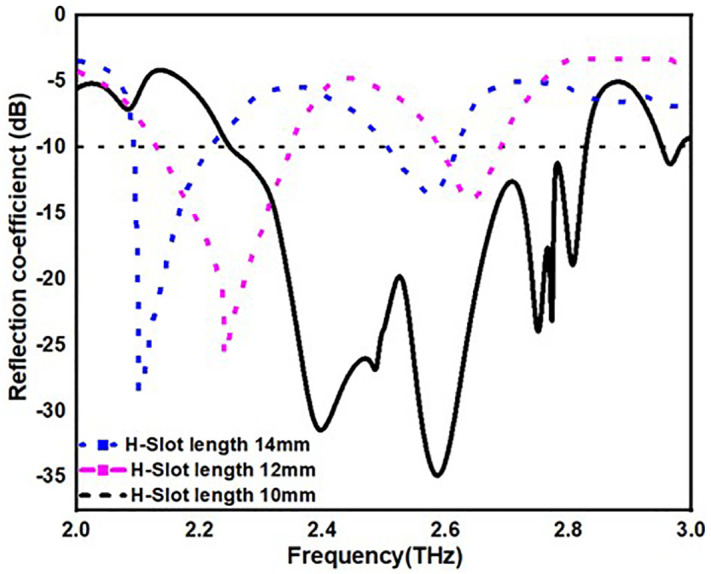
S11 value by changing substrate width.

**Fig 16:**
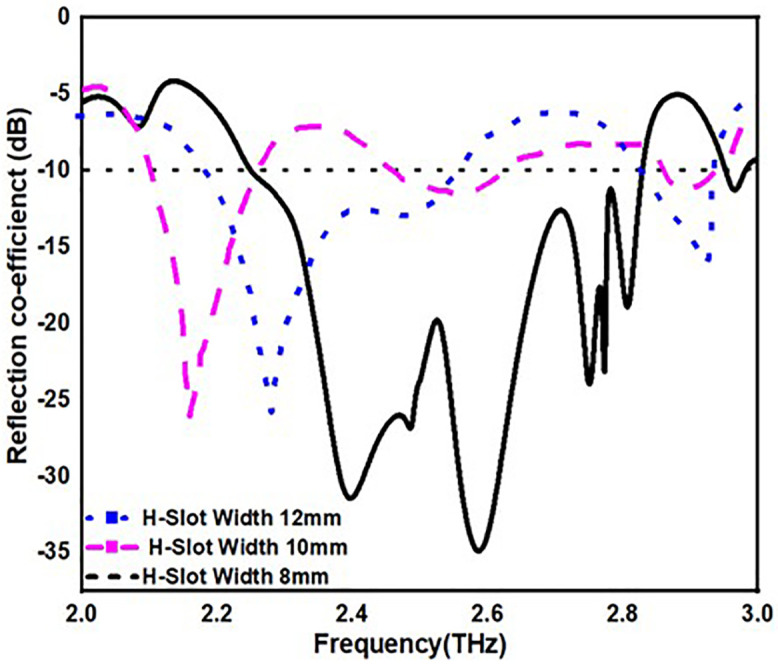
S11 value by changing patch radius.

From Figs 13–16, it is clearly visible that by changing the dimension of the antenna one can change the antenna for multiple application. From Fig 13. one can observe that by increasing the width of the substrate to 50 mm one can achieve antenna for multiple application instead of single application. Similarly, in Fig 14. by changing the radius of the circular patch from 21 mm to 19 mm we can use our antenna for various applications instead single application. In addition to this by changing the length and width of H-slot which lies in patch from 14 mm to 10 mm and 12 mm to 8 mm one can use the proposed antenna for various application with minimum return loss value in Figs 15 and 16.

To summary, parametric analysis is critical to the methodical investigation and optimisation of antenna designs, which aids in striking a balance between functionality, size, cost, and performance. it is an essential tool in contemporary antenna engineering, particularly in sectors like defence, aerospace, and telecommunications that demand high-performance antennas.

Table 4 also stated the numerical value of the parametric analysis of the proposed antenna across various dimensions of the physical parameters.

**Table 4:** Parametric analysis of the proposed antenna.

Parametric Analysis of substrate width		Parametric Analysis		Parametric Analysis		Parametric Analysis	
Dimensions (mm)	Operating Frequency, (B.W)-(THz)	Dimensions (mm)	Operating Frequency, (B.W)	Dimensions (mm)	Operating Frequency, (B.W)	Dimensions (mm)	Operating Frequency, (B.W)
48mm	2.5,2.85-(0.5,0.15)	21	2.3,2.85-(0.3,0.1)	14	2.1,2.6-(0.35,0.1)	12	2.3,2.9-(0.4,0.1)
49mm	2.2,2.6-(0.3,0.1)	20	2.2,2.5-(0.3,0.1)	12	2.3,2.7-(0.5,0.2)	10	2.1-(0.25)
50mm	2.25-2.85,(0.6)	19	2.25-2.85,(0.6)	10	2.25-2.85,(0.6)	8	2.25-2.85,(0.6)

## 3. Results and discussion

Results for different antenna settings were obtained by analysing the configuration shown in Fig 2. Initially a parametric analysis was performed to obtain the optimization of the device by varying the geometry of the proposed design. Later, ones the optimization was completed the antenna parameters was evaluated in terms of its reflection co-efficient, insertion loss, radiation patters and Gain value. On top of it, the antenna is a MIMO antenna so therefore MIMO parameters also evaluated in the section like ECC, MEG, DG, CCL and TARC. For the Proposed Antenna the results obtained are in good condition in order to applicable for real time application.

**Fig 17:**
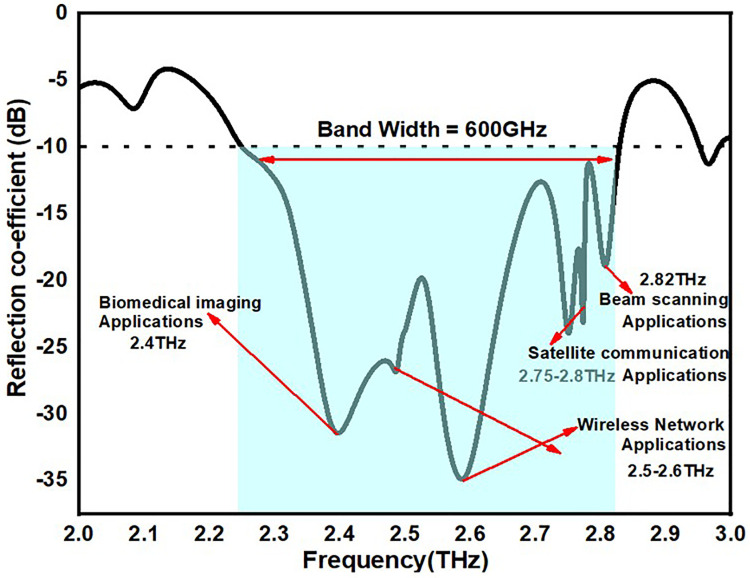
Reflection coefficient value of the proposed MIMO antennas.

The suggested antenna’s reflection coefficient value over the operating frequency is shown in Fig 17. According to the suggested antenna, the operational frequency spans 2.25 THz to 2.85 THz, or 0.6 THz of bandwidth. These antennae also operate across Biomedical imaging applications, wireless network applications, Beam scanning applications as well as satellite communication applications with a reflection co-efficient values < -25dB.

**Fig 18:**
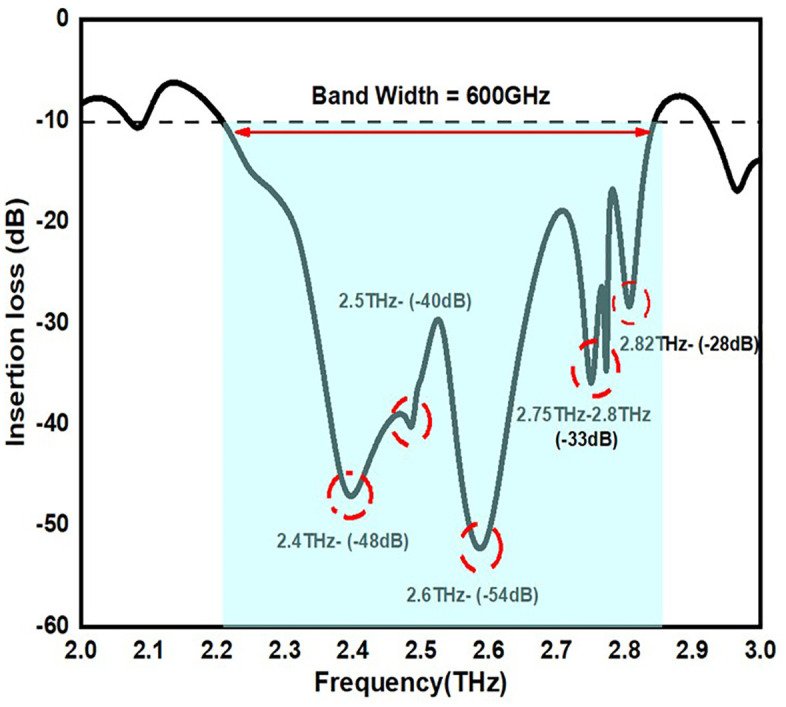
Insertion loss value of the proposed MIMO antennas.

When building a MIMO antenna, mutual coupling is a crucial element since it may result in a number of impacts, including signal correlation, impedance mismatch, and distortion of the radiation pattern. So, finally in order to minimize this effect in the proposed antenna we maintained an optimum distance between the two antennas. In addition to this optimum dimension of the antenna and implementation of the ground plane also performed to minimize the mutual coupling. The Fig 18 states the insertion loss value of the proposed antennas. The antenna states the insertion loss value of <-40dB across the operating frequency. From the Fig 18 it is visible that minimum the insertion loss value minimum the mutual coupling effect.

Table 5 states insertion loss value of the proposed antenna across two ports in detail. By seeing the Table 5. It is clearly evident that across two ports the device showing almost similar values.

**Table 5:** Insertion loss value of the proposed antenna across Port 1 and Port 2.

Operating Frequency (THz)	Port 1	Port 2
2.4	-48dB	−48.65dB
2.5	-40dB	−39.75dB
2.6	-54dB	−52.45dB
2.75	-33dB	−33.6dB
2.82	-28dB	−26.78dB

**Fig 19:**
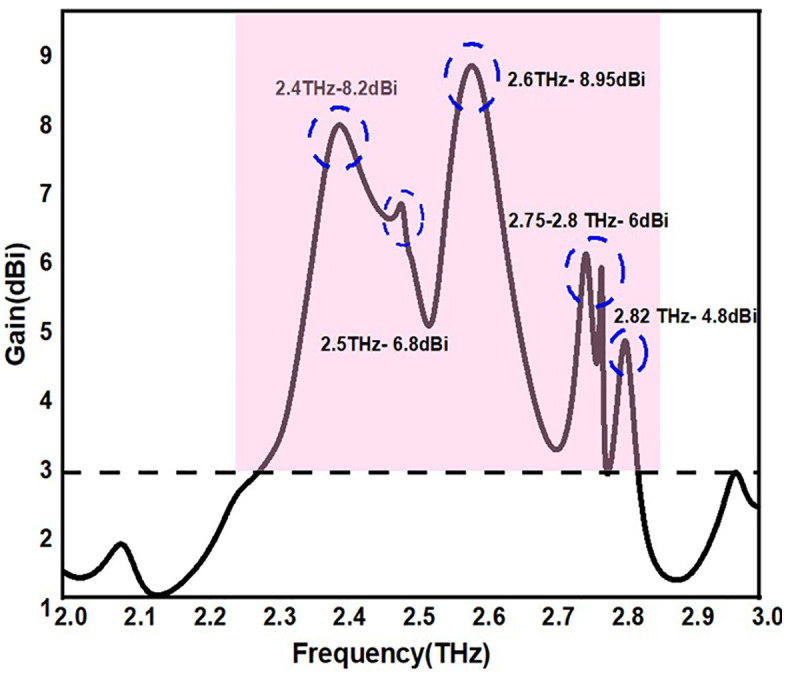
2D-Gain patterns of the proposed antennas.

The antenna gain is an important parameter to strength the signal of the device, to improve the directivity of the device. In addition to this to enhance the network capacity and efficient power usage gain of the antenna place an important role. The suggested antenna’s 2D gain value is shown in Fig 19. The antenna has a 4.8dBi to 8.9dBi gain value that spans the operational frequency with a 0.6 THz bandwidth.

### 3.1 MIMO antenna performance parameters

Many wireless communication systems employ antenna MIMO system to improve spectrum efficiency, data throughput, and connection stability. The following are important MIMO antenna performance parameters: When building a MIMO antenna, mutual coupling is a crucial element since it may result in a number of impacts, including signal correlation, impedance mismatch, and distortion of the radiation pattern.

#### 3.1.1 Total Active Reflection Co-efficient (TARC).

All of the ports in a MIMO antenna are activated simultaneously when amplitude and signal phase are changed. TARC is the square root of the ratio of the total incident power to the total reflected power. The TARC parameter of the antenna model is evaluated by equation 5. Fig 19. states that TARC is < 0 db. Equation (1) generate a curve by considering random values in the range between 0 and 2π.


$$TARC=\sqrt{\frac{{\left(S_{mn}+S_{mn}\right)}^{2}+{\left(S_{mn}+S_{mn}\right)}^{2}}{\text{2}}}$$ (5)

Where S states the scattering parameter value of the device Smm and Snn states reflection co-efficient value of the device. Similarly, Smn and Snm are the insertion loss design device.

**Fig 20:**
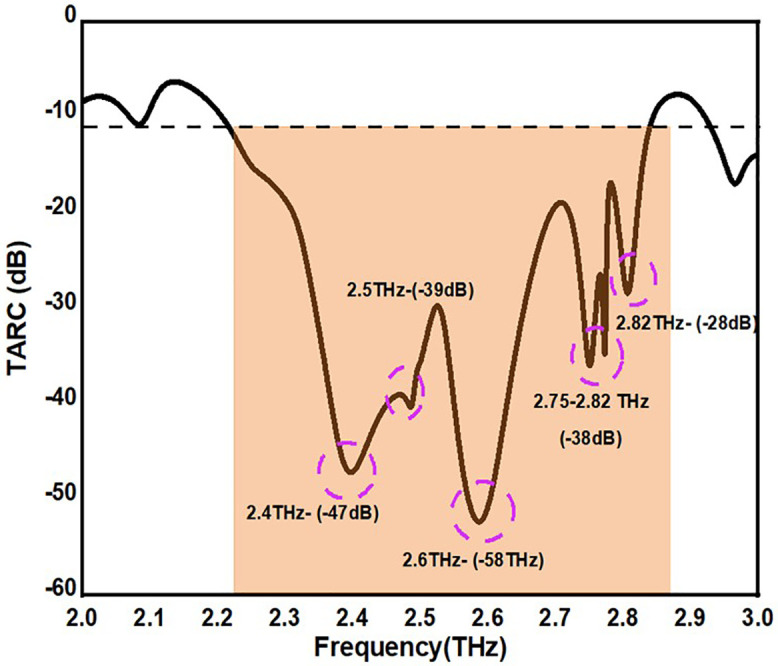
TARC Value of the proposed antennas.

The suggested antenna TARC value is shown in Fig 20. The TARC value of the suggested antenna is between -45dB and -55dB over the working frequency range. The antenna’s versatile expandable features and straightforward structure make it an excellent choice for multiband applications.

#### 3.1.2 ECC (Envelope Correlation Coefficient).

Because it directly impacts system performance, the envelope correlation coefficient (ECC) is a crucial statistic in MIMO (multiple-input multiple-output) antenna systems. It is impossible to exaggerate the significance of ECC in MIMO antenna systems. To fully benefit from MIMO technology, which includes higher data speeds, better coverage, enhanced signal quality, and more effective use of the spectrum, a low ECC is essential. Therefore, in the design and implementation of MIMO antennas for contemporary wireless communication systems, minimising ECC is a crucial factor. ECC should be zero in the ideal case and 0.5 in the tolerable case. By leveraging the far-field data, the ECC computation can be performed with more accuracy. However, the S-parameter-based equations can be employed to estimate the ECC in locations with limited resources. Equations (6) provide the ECC using far-field data and S-parameters, respectively. Fig 20. displays the ECC varying ports. The obtained values shows that ECC value is less than 0.5.


$$ECC=\frac{|S_{mn}^{*}S_{mn}+S_{mn}^{*}S_{nn}|}{(1-||{S_{mn}^{*}|}^{2}-|{S_{mn}^{*}|}^{2})-(1-|{S_{mn}^{*}|}^{2}-|{S_{mn}^{*}|}^{2}{)}^{*}}$$ (6)

**Fig 21:**
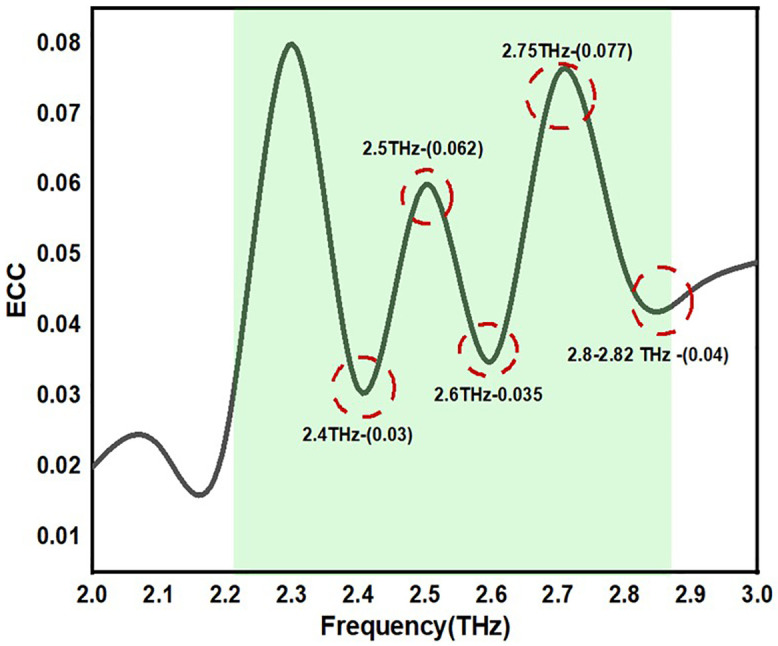
ECC of the proposed antennas.

Fig 21 states the ECC value of the proposed antennas. The antenna states the ECC value of 0.03 to 0.08 range across the operating frequency. The proposed design is of simple structure and flexible extensible characteristics which enhances the design for multiband applications.

#### 3.1.3 MEG (Mean Effective Gain).

MEG is defined as the average gain of an antenna by considering power density of the incoming signals in all directions. It is a statistical measure that takes into account the characteristics of the surrounding signal as well as the emission pattern of the antenna. Mean Effective Gain (MEG) is an important statistic in the design and study of MIMO antennas. It sheds light on the efficient ways in which antennas may pick up signals in a variety of settings and orientations. In order to achieve balanced performance, maximise capacity, and guarantee reliability in MIMO systems, it is imperative to comprehend and optimise MEG. Equation (7) is used to calculate the mean effective gain. The numbers that were determined numerically are displayed in Table. The practical value specified for the proposed antenna is < −3 less than or equal to MEG (dB) <−12 for good performance. As a result, all MIMO designs’ MEG values were validated.


$$\begin{array}{c} MEG_{i}=0.5\mu_{irad}=0.5(1-\sum_{j=1}^{K}|S_{ij}|) \end{array}$$ (7)

Where i= antenna represents under observation, µ= radiation efficiency

**Fig 22:**
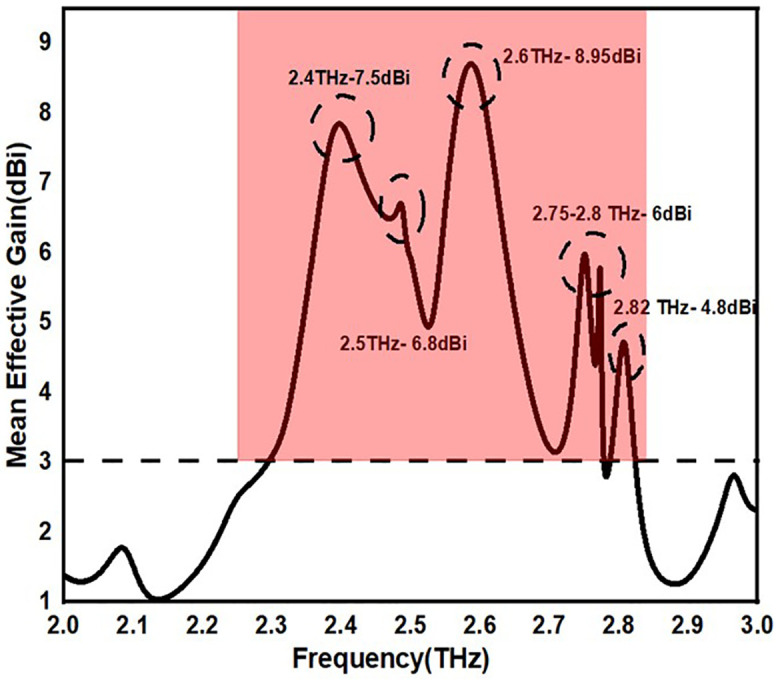
MEG of the proposed antennas.

Fig 22 shows the MEG values of >7.5 and <9 dBi over the operating frequency for the proposed antenna. Despite its straightforward structure and adaptable expandable options, the proposed antenna design improves the design for multiband applications.

#### 3.1.4 CCL (Channel Capacity Loss).

Multiple-input channel capacity The greatest data rate possible when employing several receiving and transmitting antenna at the same time is known as multiple-output (MIMO) systems. Modern wireless communication systems, like Wi-Fi, 4G, and 5G networks, heavily depend on MIMO technology due to its ability to significantly increase spectral efficiency and data throughput. In MIMO systems, channel capacity loss is very significant for a number of reasons: like network planning and optimization, minimize the system design and its complexity, maximize the quality of the service, minimize the signal interference and improves the correlation between the channel. In addition to this MIMO systems may be more susceptible to jamming and eavesdropping assaults if their channel capacity is smaller. Ensuring the security and resilience of wireless communication networks requires optimising channel capacity.

**Fig 23:**
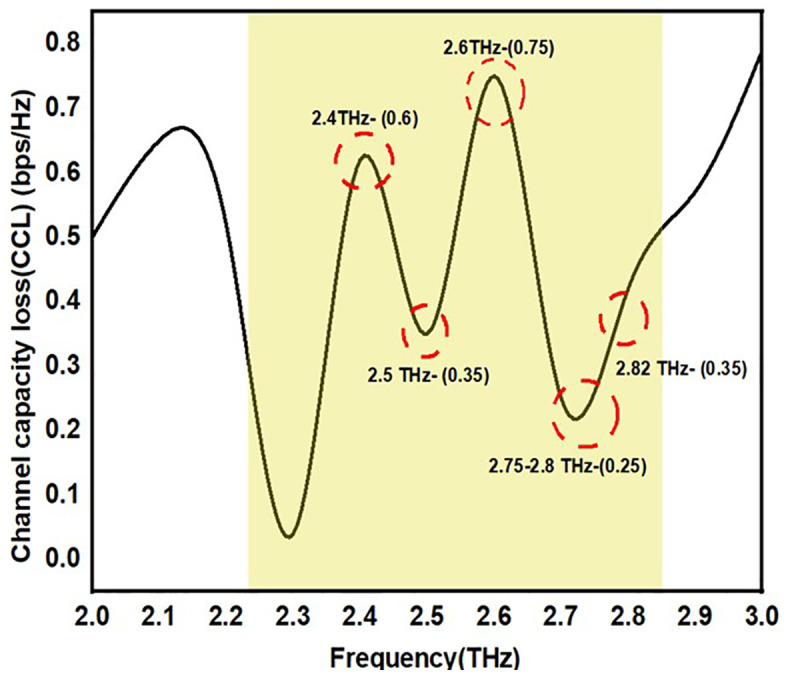
CCL value of the proposed antenna.


Therefore, by providing the details of channel capacity losses during correlation effect. CCL is evaluated numerically using the formulas (5–8). For the whole operating band, CCl is set to the usual value of 0.4-bit/s/GHz.The design is validated with the performance. To determine the rate of information delivered consistently via the communication channel, channel capacity loss (CCL) is required. The following equations (5–8) are used to determine the CCL. The CCL of the suggested MIMO antenna is shown in Fig 23. It is observed that the values of CCL are below the threshold level of standard value.

$$C(loss)=-\log_{2}\det(a)$$ (8)

$$a=\left[\begin{array}{c} \sigma_{11}\sigma_{11}\\ \sigma_{21}\sigma_{21} \end{array}\right]$$ (9)

$$\sigma_{ii}=1-({|S_{ii}|}^{2}-{|S_{ii}|}^{2})$$ (10)

$$\sigma_{ij}=-({S_{ii}}^{*}S_{ij}+{S_{ji}}^{*}S_{jj})$$ (11)

The CCL value of the suggested antennas is shown in Fig 23. CCLs of 0.25 to 0.75 are reported by the antenna over the entire working frequency range. Using the suggested antenna design improves multiband applications due to its straightforward structure and adaptable features.

#### 3.1.5 Directivity Gain (DG).

In MIMO systems, it is especially important to factor in antenna directivity gain. It refers to the ability of an antenna to focus energy in a single direction, improving signal strength and communication quality in that direction. Directivity gain is a key factor in improving the overall performance of MIMO systems, which use multiple antennas at the transmitter and receiver. These benefits include improved signal strength, signal-to-noise ratio (SNR), spatial multiplexing gains, interference management, coverage and capacity, optimized beamforming, and energy efficiency. Using equation, the diversity gain is computed (12).


$$DG=10\sqrt{1-|{\rho_{e_{ij}}|}^{2}}$$ (12)

**Fig 24:**
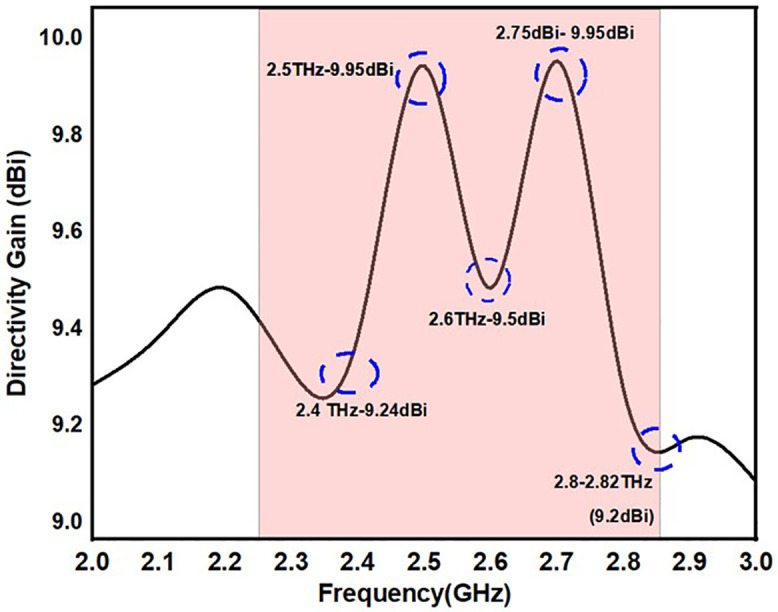
DG value of the proposed antenna.

Fig 24 states the DG value of the proposed antennas. According to the antenna, the DG value ranges from 9.2 to 9.95 over the operational frequency. The design of the proposed antenna offers improved performance for multiband applications due to its straightforward structure and adaptable, expandable features.

### 3.2 Radiation patterns

In wireless communication systems, the radiation pattern of an antenna is essential to its functionality. It impacts every aspect of life, including energy efficiency, security, interference control, and signal strength and range. Understanding and optimising radiation patterns is essential to building efficient, dependable, and effective antennas that satisfy the unique needs of their intended applications.

**Fig 25:**
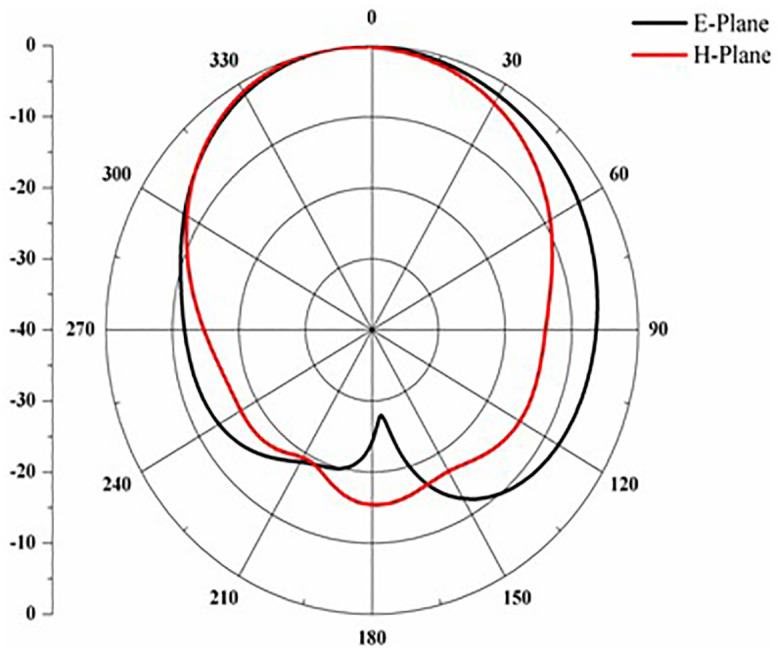
DG value of the proposed antenna.

Fig 25 states the DG value of the proposed antennas. According to the antenna, the DG value ranges from 9.2 to 9.95 over the operational frequency. The design of the proposed antenna offers improved performance for multiband applications due to its straightforward structure and adaptable, expandable features Fig 25. E-H plane across 2.4 GHz.

**Fig 26:**
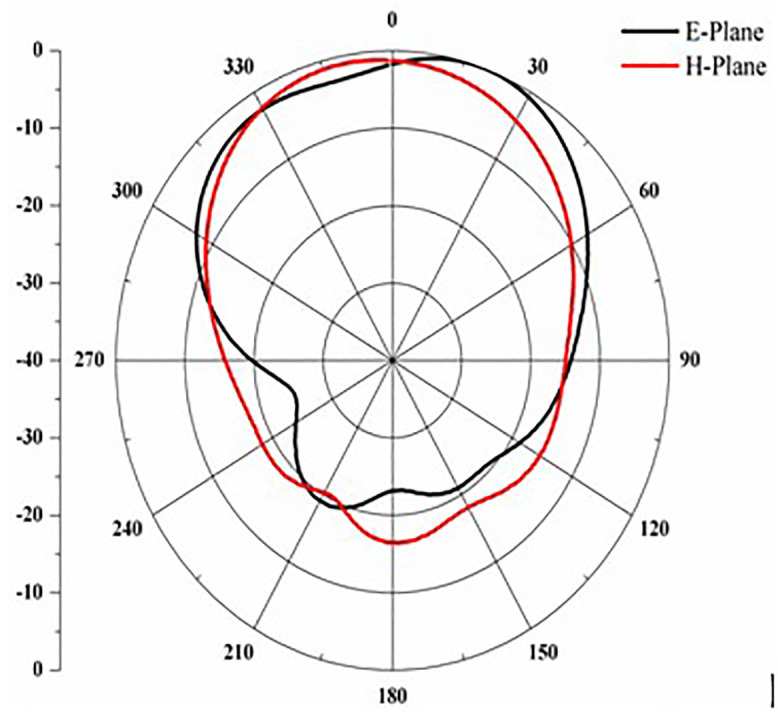
E-H plane across 2.5 GHz.

**Fig 27:**
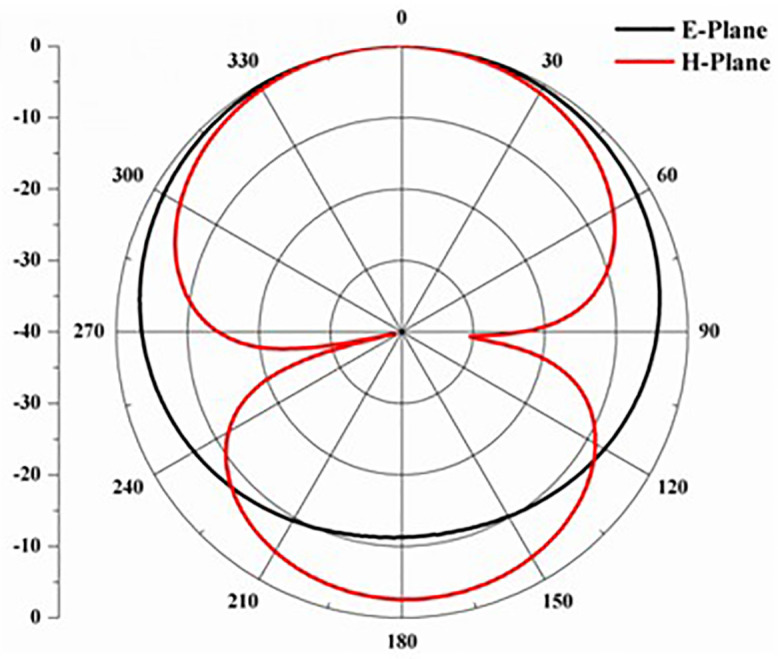
E-H plane across 2.6 GHz.

**Fig 28:**
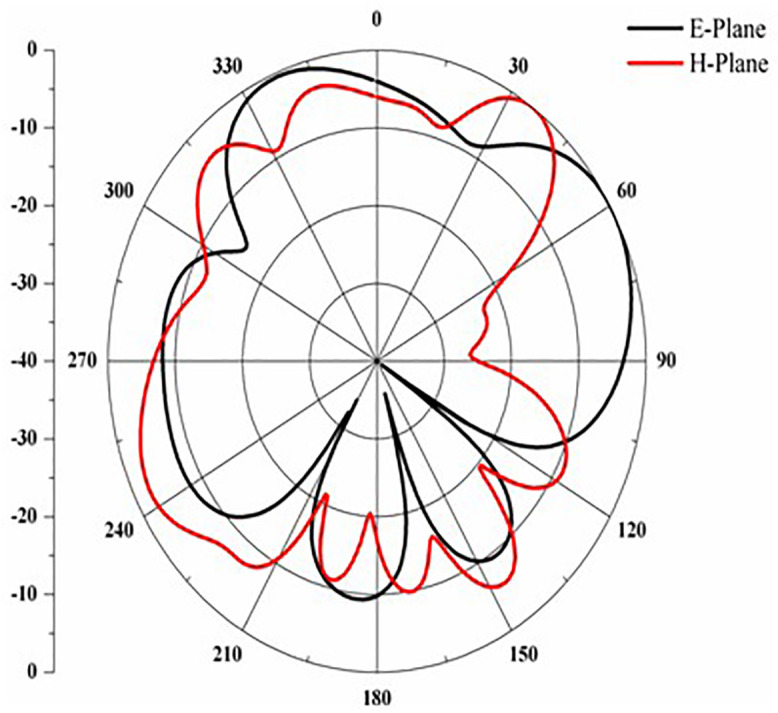
E-H plane across 2.75 GHz.

**Fig 29:**
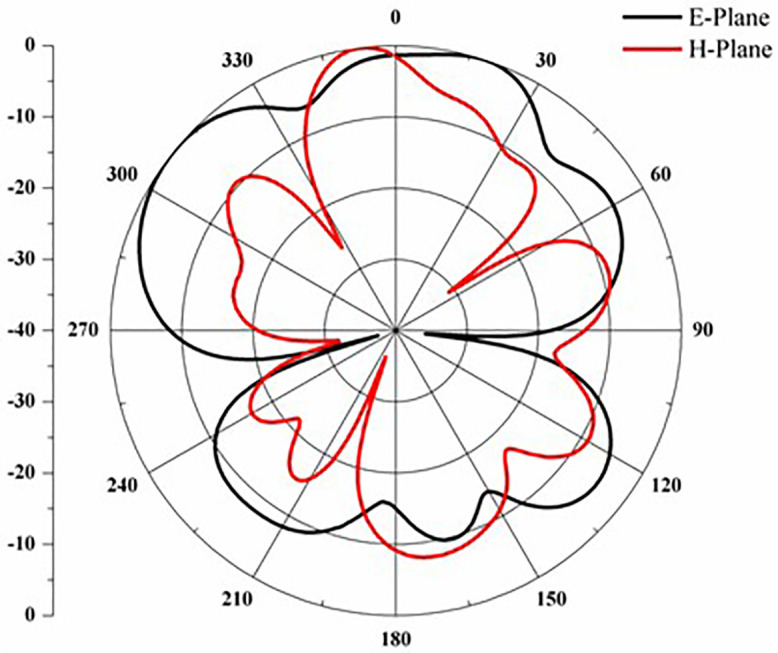
E-H plane across 2.82 GHz.

**Fig 30:**
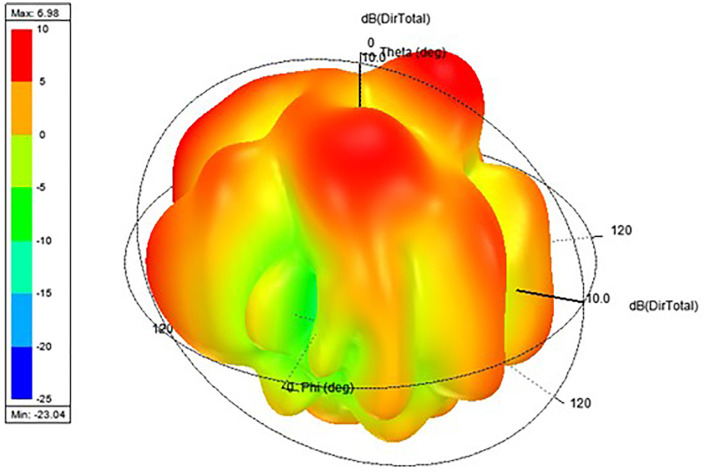
3D Radiation pattern E-H plane across 2.42 GHz.

**Fig 31:**
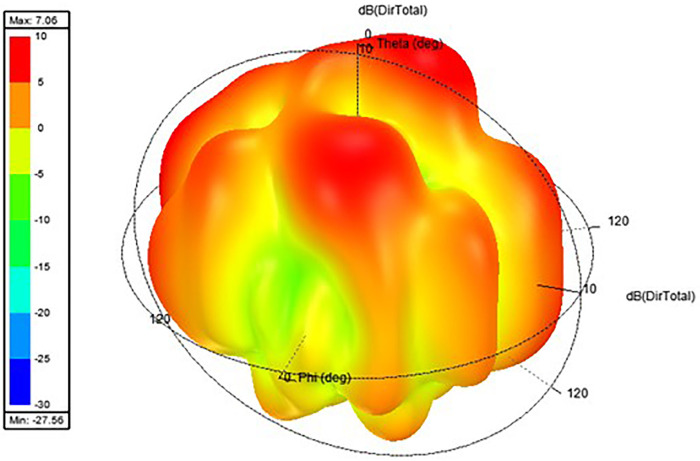
3D Radiation pattern E-H plane across 2.8 GHz.

Proposed antenna radiation patterns are stated in Figs 25 and 26. Throughout the operational frequencies, the antenna exhibits an omnidirectional radiation pattern in both the e and h planes. Similarly, Fig 27 states E-plane in omnidirectional and H-plane in dumbbell shape. In addition to this Figs 28 and 29 states E-plane in maximum top lobe and dumbbell shape along with H-plane in butterfly shape across the operating frequency with a angle of elevation of 0 deg and 90 deg. Figs 30 and 31 states the 3D radiation pattern values of the proposed antenna across the operating frequency value of 2.4 GHz and 2.8 GHz with a value of 6.98 and 7.05dB.

### 3.3 Surface currents distribution

In order for a MIMO antenna system to operate optimally, the surface current distribution must be correct. It affects important factors such mutual coupling, impedance, radiation patterns, efficiency, and the system’s capacity to handle large data rates with little interference. For current communication systems, designing effective, high-performance MIMO antennas requires an understanding of and control over this distribution.

**Fig 32:**
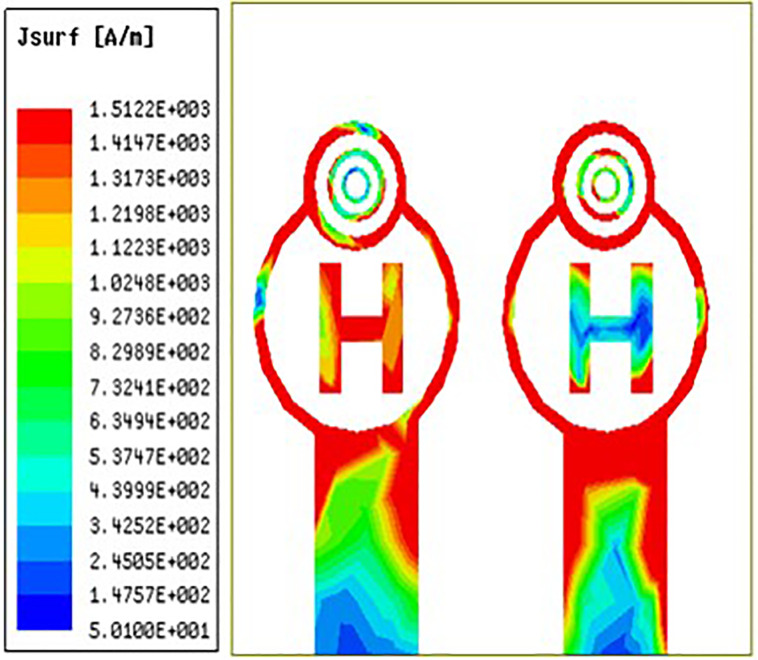
Least surface currents distribution.

**Fig 33:**
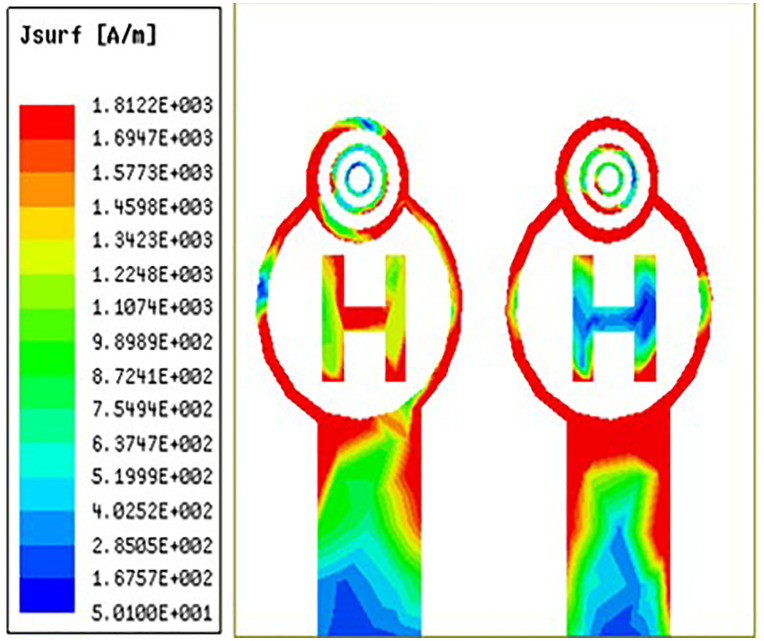
Mid-range surface currents distribution.

**Fig 34:**
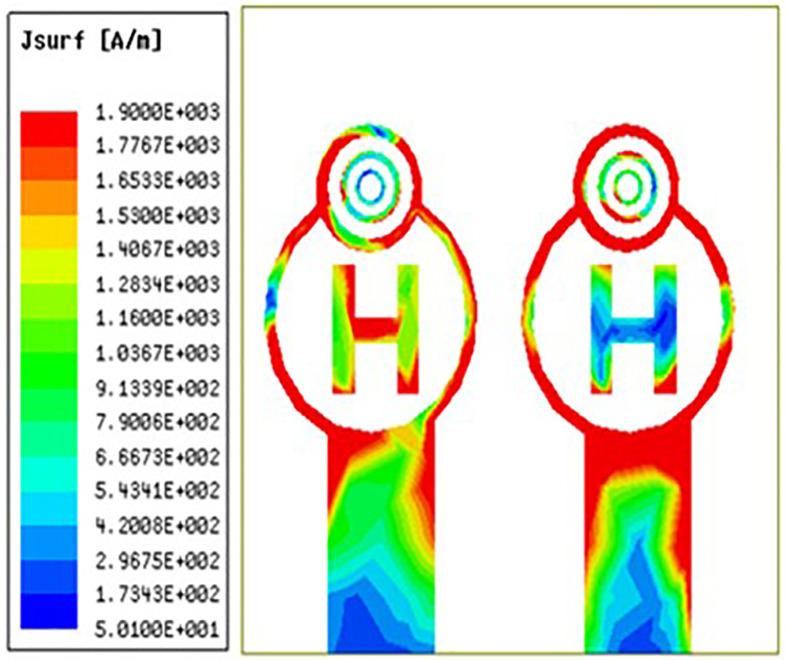
High-range surface currents distribution.

From Figs 32–34 it is clearly visible that as the frequency shifts from left to right the surface current are increased from left to right. The red colour indicates maximum surface current distribution as well as the blue colour indicates minimum surface current distribution. Across the circular ring and H-Slot the antenna states maximum surface currents and on other side of the coin across the feed the antenna states average surface current distribution. From Fig 32 the antenna states surface current distribution value of 152 A/m. Similarly, Figs 33 and 34 the antenna states surface current distribution value of 181 A/m and 190 A/m.

### 3.4 E-field distribution

The electric field distribution must be taken into account when optimizing MIMO antenna performance, including radiation pattern management, impedance matching, mutual coupling, polarisation, efficiency, gain, and bandwidth. It is important to understand and engineer the electric field distribution accurately in MIMO systems, particularly when sophisticated techniques like beamforming and spatial multiplexing are involved.

**Fig 35:**
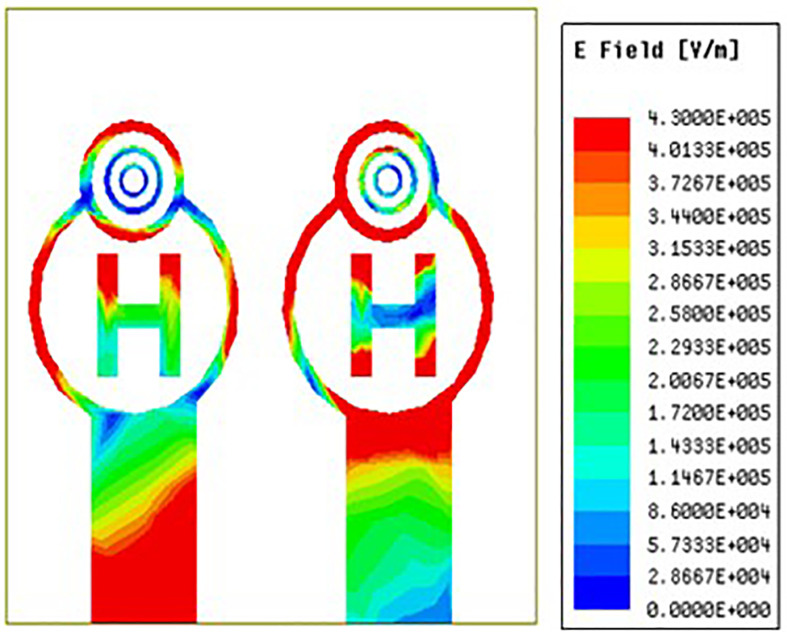
Least E-feild distribution.

**Fig 36:**
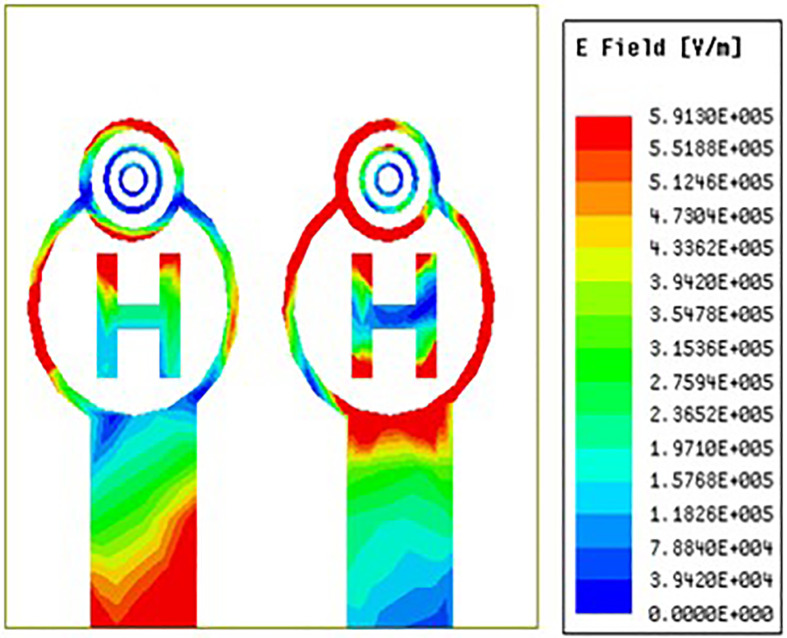
Mid-range E-feild distribution.

**Fig 37:**
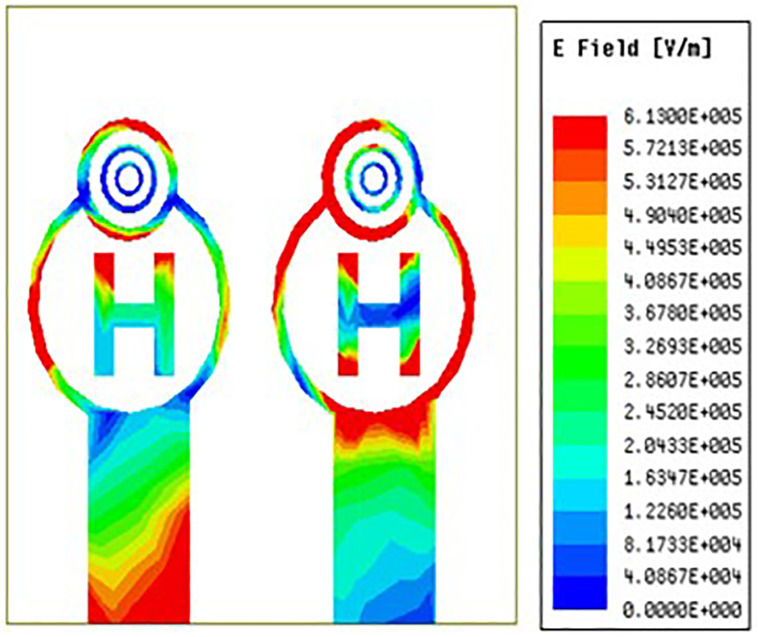
High-range E-feild distribution.

Figs 35–37 makes it very evident that the E-field distribution increases from left to right as the frequency moves from left to right. Both the maximum and minimum E-field distributions are indicated by the colours red and blue, respectively. Across the circular ring and H-Slot the antenna states maximum E-field and on other side of the coin across the feed the antenna states average E-field distribution. From Fig 35 the antenna states E-field distribution value of 4.9e^5 V/m. Similarly, Figs 36 and 37 the antenna states E-field distribution value of 5.91e^5 V/m and 6.13e^5 V/m.

### 3.5 H-field distribution

For MIMO antennas to provide optimal bandwidth, regulate mutual coupling, achieve adequate impedance matching, and ensure efficient radiation, the distribution of magnetic fields is crucial. It affects the radiation patterns, isolation, efficiency, and polarisation of the antenna. Achieving optimal performance in MIMO systems necessitates careful consideration and management of the magnetic field distribution, particularly in scenarios where densely packed antennas are needed or where certain beamforming and diversity properties are needed.

**Fig 38:**
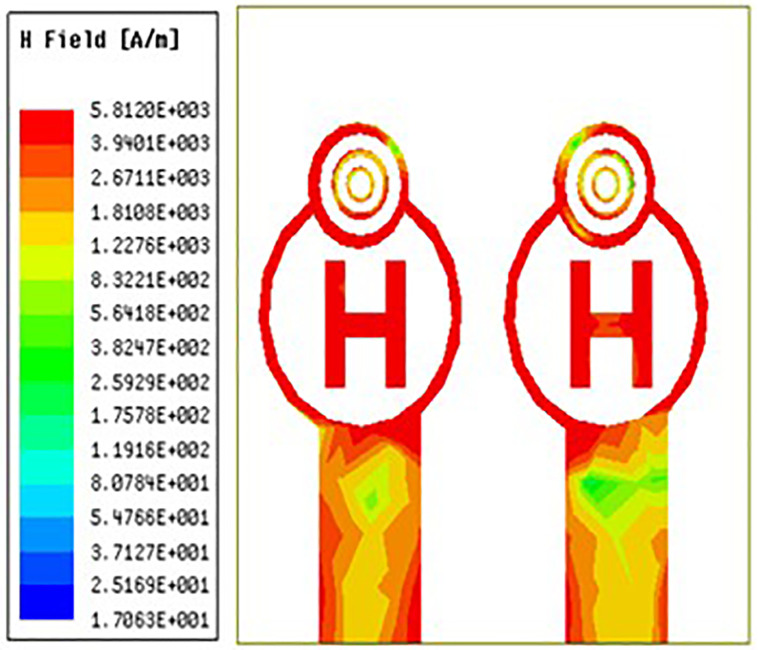
Least H-feild distribution.

**Fig 39:**
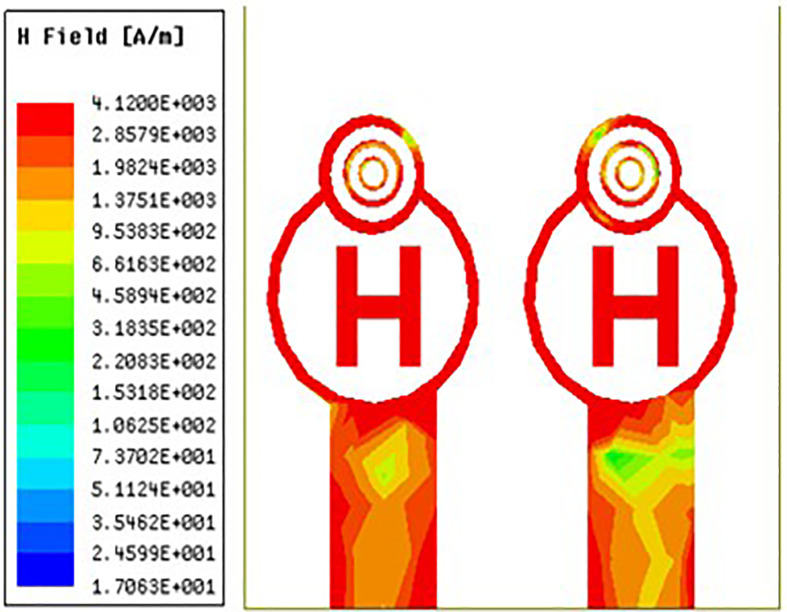
Mid-range H-feild distribution.

**Fig 40:**
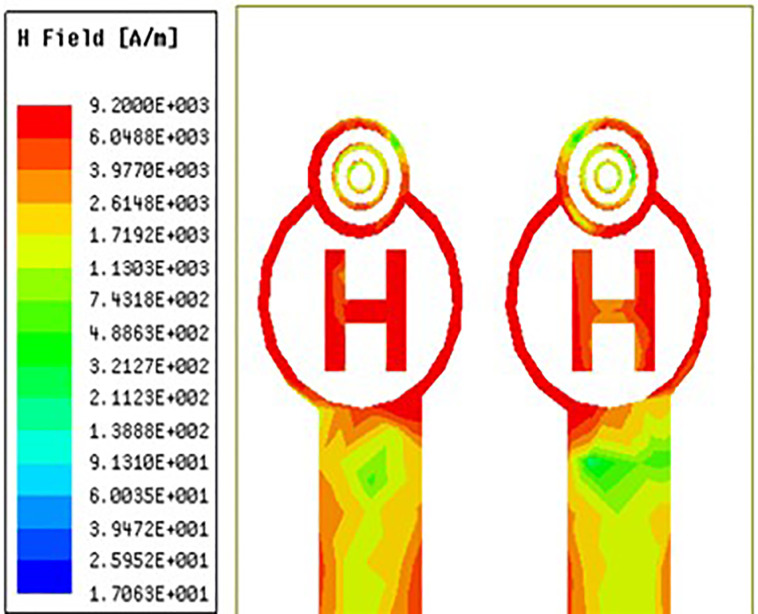
High-range H-feild distribution.

Figs 38–40 makes it very evident that the H-field distribution increases from left to right as the frequency moves from left to right. Both the maximum and lowest H-field distributions are indicated by the colours red and blue, respectively. The antenna displays the highest H-field throughout the circular ring and H-Slot, and the average H-field distribution over the feed on the other side of the coin. Its H-field distribution value is 581 A/m in Figs 38–40 illustrate the antenna’s H-field distribution values of 412 A/m and 920 A/m, respectively.

The frequency bands are now open up to 3 THz for experimental licensing as authorized by the Federal Communications Commission (FCC) [17]. The main feature of the THz is high polarisation, which facilitates the detection of opaque objects and may have security implications. THz communication also offers very little transmission loss and no danger. Better spatial resolution and high spectrum information are also provided. The beams of THz waves are well directed. THz waves may thus be the best option for very high-speed wireless networks [25,26]. Table 6 provides a comparison of 4G and 6G technologies.

**Table 6:** Fourth Generation, Fifth Generation and Sixth Generation Comparison.

Parameters	Fourth Generation	Fifth Generation	Sixth Generation
Operating frequency range	1900–2100 MHz, 2200–2400 MHz	2-8,24–100 Giga Hertz	0.1-10 Tera Hertz
Peak information rate	100 Mega bite Pico seconds	10 Giga bite Pico seconds	1 Tera bite Pico seconds
End – to-end (E2E) dormancy	Ten milli seconds	One milli second	10-100 micro seconds
Peak otherworldly effectiveness	15 bite Pico seconds/ Hertz	30 bite Pico seconds/ Hertz	60 bite Pico seconds/ Hertz
Advanced Automation	MIMO, OFDM	Massive MIMO, milli meter wave	Spatial modulation MIMO, Tera hertz Communication
Network stage	Packet internet protocol	Cloud technologies, Partial artificial intelligence, Machine learning	Artificial intelligence and Machine learning
Use situation	MBB	Massive machine type communication (mMTC), and ultra-reliable low latency communication (URLLC)	Massive ultra-reliable low-latency communication (mULC), (ULBC)
Characterizing application	World wide web	Industrial internet of thinks	Digital worlds
Portability (km/h)	350	500	1000
Client experienced information rate	20 Mbps	0.1-1 Giga bite Pico seconds	1 Giga bite Pico seconds
Range of transmission	5 km	100 plus meter	10 plus meter
Real time Applications	Digital payment, Mobile internet and HD videos	UHD videos, wearable technology, and smart cities	Augmented and virtual reality (AR/VR), fully automated driving, industrial internet, internet of bio nano-things

### 3.6 Total efficiency of the proposed antennas

One important factor affecting an antenna’s performance is its radiation efficiency. It shows how well an antenna transforms incoming power into electromagnetic waves that are radiated, as opposed to losing it as heat or through other types of loss. Its primary applications include maximising power utilisation, reducing heat and material degradation, improving signal-to-noise ratio (SNR), enhancing communication range, conserving energy in power-sensitive applications, and minimising interference. To sum up, radiation efficiency is critical to obtaining the best results in every application that uses antennas, from lowering interference and system wear to increasing communication range and power economy.

**Fig 41:**
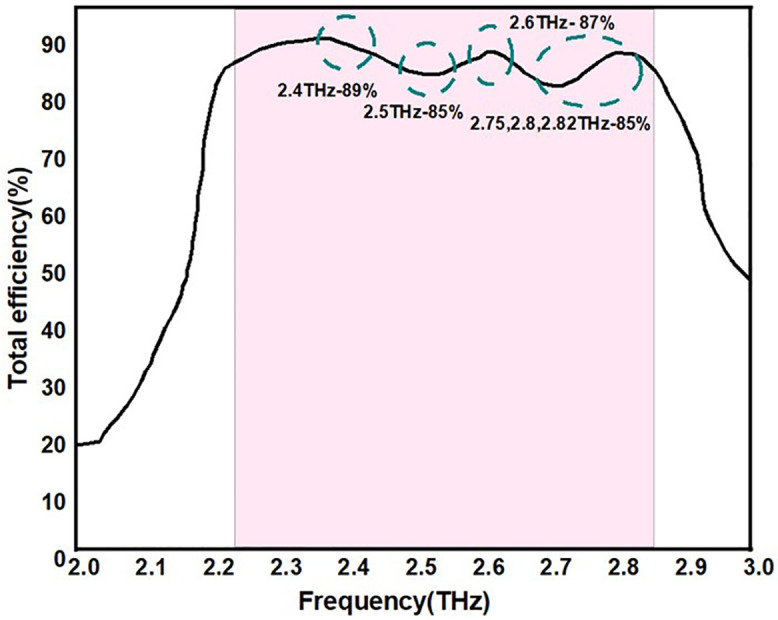
Total radiation efficiency of the proposed antennas.

From Fig 41 it is clearly observed that the antenna showing high radiation efficiency value range between 85–89% which is highly acceptable value across operating frequencies.

### 3.7 Equivalent circuit of the proposed antenna

**Fig 42:**
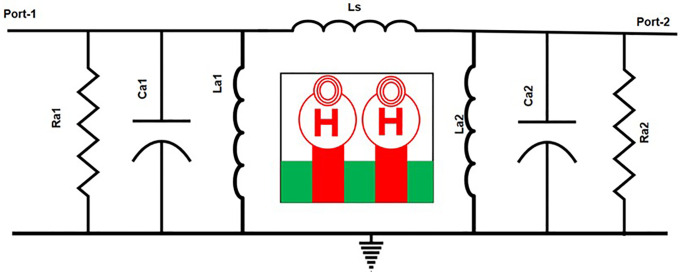
Equivalent circuit of the proposed MIMO antenna.

This Fig 42 illustrates a two-port equivalent circuit model representing a coupled antenna configuration. Each port contains resistive (Ra), capacitive (Ca), and inductive (La) components that characterize the antenna’s impedance behavior. The central inductor (Ls) models the mutual coupling between the two antennas. Overall, this circuit is used to study parameters such as mutual coupling, bandwidth, and isolation in antenna arrays or MIMO systems.

### 3.8 SAR (Specific Absorption rate if the proposed MIMO antenna)

**Fig 43:**
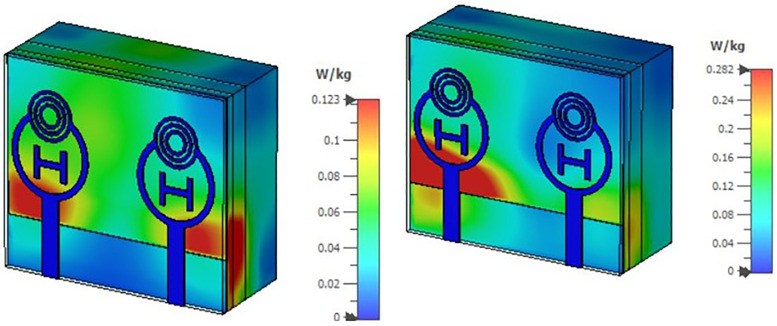
SAR analysis across Bio-medical frequency (2.85 GHz) of the proposed MIMO antenna.

SAR (Specific Absorption Rate) measures how quickly biological tissue absorbs electromagnetic energy when exposed to an EM field. The two images likely depict different antenna setups, polarizations, or input power levels at 2.85 THz. The colour gradient (blue to red) in Fig 43. represents absorption intensity: blue/green indicates low SAR (minimal tissue heating), while yellow/red shows higher SAR (greater localized absorption). The second configuration exhibits increased energy absorption, which may result from stronger field concentration near the tissue, closer antenna-tissue interaction, or higher input power. Both SAR values remain well below the safety thresholds established by IEEE C95.1 and ICNIRP (commonly 2 W/kg averaged over 10 g of tissue). This confirms that the 2.85 THz antenna is safe for biomedical applications such as non-invasive imaging, sensing, or communication.

From the Table 7, clearly observed that the proposed design has a minimum dimension having an ECC value lower than other works with a minimum insertion loss value with a wide bandwidth of 600 GHz. The proposed design will be used for various applications like Biomedical imaging applications, wireless network applications, Beam scanning applications as well as satellite communication applications. Similarly, the proposed antenna has high gain values of 4.8–8.95 dB than the other literature in the Table 6.

**Table 7:** Comparison of the proposed antenna with existing literature survey.

Ref. no.	Dimensions (μm3)	Operating frequency (THz)	No.of ports	Design of antenna	Gain (dBi)	Isolation (dB)	ECC	Application
[25]	800x1170x5	2.2,3.2,4.5	2&8	THz graphene base MIMO antenna array	5.8	>15	<0.2	Tera hertz MIMO communication
[26]	60x40x1.6	1.76–1.87	2 & 1	MIMO antenna graphene based	4.45	>25	<0.5	Communication devices in future
[27]	67.5x67.5x1.6	1.5-1.0	4	Four element MIMO antenna	11	20	<0.5	Aerospace uses and deep space networks
[28]	800x1170x81.29	0.1-1.5	2&2	Tetradecagon ring-shaped MIMO antenna array	–	30	> 0.5	Tera hertz short distance communications
[29]	80x60x0.1	1.291	2	MIMO antenna	–	<30	>0.5	Wireless communications
[30]	65x65x1.6	8.84	2&6	MIMO antenna	8	22.26	<0.7 and>0.5	WBAN
[31]	1000x1400x1.29	0.33-10	2&2	THz super wide band antenna	5.2	22	0.15	Applications in astronomy, THz wave radar, 3D printing, automobile communications, and healthcare
[32]	43x59x320	1.24	2	THz antenna with copper substrate	4	20	>0.5	Wireless communications
Proposed work	50x50x100	2.25-2.82	2	Tetradecagon ring-shaped MIMO antenna array	4.8-8.95	>28 and <54	<0.08	Biomedical imaging applications, wireless network applications, Beam scanning applications as well as satellite communication applications.

Fabricating terahertz antennas is challenging due to several factors:

The very small physical dimensions necessitate precise micrometre-scale manufacturing techniques. High losses in materials and conductors demand careful selection of substrates and metals.

Multilayer and on-chip integration increase complexity in alignment and assembly.

Tight fabrication tolerances make the antennas extremely sensitive to even minor errors.

Characterizing and testing these antennas at THz frequencies is difficult and requires specialized, resource-intensive equipment.

## 4. Conclusion

Ultra-high data rates must be supported by future wireless communication systems; THz band communication will be essential to reaching this objective. Achieving extremely high data speeds requires high-gain antennas. The proposed antenna has circular rings and H-shaped slots across the patch of the original design to enhance the device’s performance. The proposed design is compared to a simple patch antenna to show performance improvement. The proposed antenna has an isolation loss value of 50dB over the operating frequency and a bandwidth of 0.6 THz. The antenna with the highest gain value in the proposed design is 8.95 dBi. Parametric optimisation with geometrical parameters is used to optimise the antenna. The proposed antenna has the following dimensions: 50x50x100. Due to its high gain and operating frequency applicability, the proposed antenna may be used for high-speed communications. The suggested antenna has an optimal TARC value of less than 30, a low ECC value of less than 0.08 and a high DG value with little mutual coupling.
